# RCAN1 knockout and overexpression recapitulate an ensemble of rest-activity and circadian disruptions characteristic of Down syndrome, Alzheimer’s disease, and normative aging

**DOI:** 10.1186/s11689-022-09444-y

**Published:** 2022-05-24

**Authors:** Helen Wong, Jordan M. Buck, Curtis Borski, Jessica T. Pafford, Bailey N. Keller, Ryan A. Milstead, Jessica L. Hanson, Jerry A. Stitzel, Charles A. Hoeffer

**Affiliations:** 1 Institute for Behavioral Genetics, University of Colorado Boulder, 1480 30th Street, Boulder, CO 80309-0447 USA; 2 Department of Integrative Physiology, University of Colorado, Boulder, CO 80303 USA; 3 Linda Crnic Institute, Anschutz Medical Campus, Aurora, CO 80045 USA

**Keywords:** Down syndrome, Alzheimer’s, Calcineurin, Circadian rhythms, DSCR1, Free-running, Light-entrained, Aging

## Abstract

**Background:**

*Regulator of calcineurin 1* (*RCAN1*) is overexpressed in Down syndrome (DS), but RCAN1 levels are also increased in Alzheimer’s disease (AD) and normal aging. AD is highly comorbid among individuals with DS and is characterized in part by progressive neurodegeneration that resembles accelerated aging. Importantly, abnormal RCAN1 levels have been demonstrated to promote memory deficits and pathophysiology that appear symptomatic of DS, AD, and aging. Anomalous diurnal rest-activity patterns and circadian rhythm disruptions are also common in DS, AD, and aging and have been implicated in facilitating age-related cognitive decline and AD progression. However, no prior studies have assessed whether RCAN1 dysregulation may also promote the age-associated alteration of rest-activity profiles and circadian rhythms, which could in turn contribute to neurodegeneration in DS, AD, and aging.

**Methods:**

The present study examined the impacts of RCAN1 deficiency and overexpression on the photic entrainment, circadian periodicity, intensity and distribution, diurnal patterning, and circadian rhythmicity of wheel running in young (3–6 months old) and aged (9–14 months old) mice of both sexes.

**Results:**

We found that daily RCAN1 levels in the hippocampus and suprachiasmatic nucleus (SCN) of light-entrained young mice are generally constant and that balanced RCAN1 expression is necessary for normal circadian locomotor activity rhythms. While the light-entrained diurnal period was unaltered, RCAN1-null and RCAN1-overexpressing mice displayed lengthened endogenous (free-running) circadian periods like mouse models of AD and aging. In light-entrained young mice, RCAN1 deficiency and overexpression also recapitulated the general hypoactivity, diurnal rest-wake pattern fragmentation, and attenuated amplitudes of circadian activity rhythms reported in DS, preclinical and clinical AD, healthily aging individuals, and rodent models thereof. Under constant darkness, RCAN1-null and RCAN1-overexpressing mice displayed altered locomotor behavior indicating circadian clock dysfunction. Using the *Dp(16)1Yey/+* (*Dp16*) mouse model for DS, which expresses three copies of *Rcan1*, we found reduced wheel running activity and rhythmicity in both light-entrained and free-running young *Dp16* mice like young RCAN1-overexpressing mice. Critically, these diurnal and circadian deficits were rescued in part or entirely by restoring *Rcan1* to two copies in *Dp16* mice. We also found that RCAN1 deficiency but not RCAN1 overexpression altered protein levels of the clock gene *Bmal1* in the SCN.

**Conclusions:**

Collectively, this study’s findings suggest that both loss and aberrant gain of RCAN1 precipitate anomalous light-entrained diurnal and circadian activity patterns emblematic of DS, AD, and possibly aging.

## Background

Alzheimer’s disease (AD) is a progressive neurodegenerative disorder for which the predominant risk factor is age [1]. Individuals with Down syndrome (DS) are disproportionately diagnosed with the early-age onset of AD [2], which implies that DS-associated genes may advance AD onset reminiscent of accelerated aging. While the link between these disorders has predominantly been attributed to overexpression of amyloid precursor protein (APP) that is cleaved to yield Aβ, a defining histopathological marker of AD, considerable evidence indicates a critical contribution from *regulator of calcineurin 1* (RCAN1; also known as DSCR1). Like *APP*, *RCAN1* is a Chromosome 21 (HSA21) gene overexpressed in DS due to an extra copy [3–5], but RCAN1 levels are also increased in the brains of sporadic AD patients [4–7] and normally aging individuals [7, 8]. Therefore, it is feasible that RCAN1 overexpression could contribute in part to the early-age onset of AD-linked pathology in DS.

Consistent with a causal role for RCAN1 in the age-related progression of AD, epidemiological research reveals that the rs71324311 and rs10550296 polymorphisms of *RCAN1* lower and enhance, respectively, the risk for AD diagnosis [9]. Furthermore, APP and Aβ can upregulate RCAN1 [10, 11], and RCAN1 can reciprocally induce Aβ [12] and enhance Aβ42 cytotoxicity [13]. Several studies in vitro have demonstrated that RCAN1 overexpression mediates additional AD-like pathophysiology, including tau hyperphosphorylation, mitochondrial dysfunction, oxidative stress, synaptic defects, and neuronal apoptosis [5, 14, 15]. We previously reported that neuron-specific RCAN1 overexpression in mice leads to tau pathology associated with age-dependent mitochondrial dysregulation and neurodegeneration, recapitulating hallmarks of AD [7]. Interestingly, RCAN1-overexpressing [7, 16–18] and RCAN1-null [19] mice both exhibit AD-like synaptic plasticity and memory deficits. Likewise, overexpression and loss-of-function of the *Drosophila* RCAN1 homolog *sarah* (*sra*; also known as *nebula*) both result in learning and memory deficits [20]. Taken together, these studies indicate that both upregulation and downregulation of RCAN1 may mediate aging- and early-onset AD-associated phenotypes.

The circadian clock controls not only biological rhythms but also memory [21–23] and deteriorates with age [24–27], suggesting that circadian dysfunction could be a marker of and/or a risk factor for aging-associated neurodegeneration. Rest-activity fragmentation and attenuated circadian physiological and locomotor rhythms accompany normal aging, are correlated with earlier cognitive decline, and worsen with aging-related neurodegenerative diseases including AD [28–33]. Notably, rest-activity and circadian anomalies also precede the onset of cognitive deficits in AD patients and mouse models and promote pathogenic Aβ42 accumulation [34–37]. Individuals with DS experience sleep-wake and rest-activity disturbances by childhood [38–41], intimating that diurnal and circadian activity disruptions at an early age may contribute to accelerated AD onset in DS. In support of the idea that diurnal activity and circadian dysfunction drive aging-related pathogenesis, disrupting rest-activity rhythms or the circadian clock can exacerbate and elicit both aging- [35, 42–44] and AD-like [42, 45–48] progressive neurodegeneration and cognitive decline. However, the molecular mechanisms underlying these diurnal and circadian alterations in DS, AD, and normal aging are poorly understood.

Considering that RCAN1 overexpression promotes AD-linked neurodegenerative phenotypes such as memory deficits in aged, but not young, mice [7], it is possible that RCAN1-mediated circadian dysfunction early in the course of aging may contribute to the development of cognitive impairments and AD progression. The *Drosophila* RCAN1 ortholog *sra* regulates the circadian periodicity and rhythmicity of locomotor activity as well as the expression and post-translational modification of the circadian clock proteins PERIOD and TIMELESS [49]. Similarly, the phosphatase activity of the RCAN1 substrate calcineurin (CaN) regulates clock gene expression [50]. CaN activity also exhibits daily oscillations that mediate the expression, entrainment, and phasing of circadian rhythms such as calcium channel activity in retinal photoreceptor cells [49–54]. Additionally, the RCAN1-overexpressing Ts65Dn [55, 56], Tc1 [57], and *Dp(16)1Yey/+* (*Dp16*) [58] mouse models for DS exhibit various abnormalities in diurnal rest-activity profiles, circadian period lengths, and amplitudes of circadian activity rhythms. Collectively, these findings implicate RCAN1 in diurnal rest-activity programs and circadian rhythmicity. Given that RCAN1 levels are elevated in the brains of DS, AD, and normally aging individuals [5, 7], aberrant RCAN1 signaling may disrupt circadian clock function and, in turn, promote cognitive decline and AD-related neurodegeneration. However, no prior studies have investigated if RCAN1 contributes to the age- and/or AD-related deterioration of diurnal and circadian activity rhythms.

The connections between the RCAN1 pathway, rest-activity patterns, and circadian clock function prompted us to examine diurnal and circadian activity profiles in young versus aged mice with RCAN1 overexpression and abolition. To explore the role of *RCAN1* trisomy in rest-activity and circadian abnormalities in DS, we additionally tested the hypothesis that *Rcan1* dosage correction in *Dp16* mice could restore normal diurnal rest-activity patterns or circadian activity rhythms in the DS mouse model. The present study characterizes, for the first time, the age-dependent consequences of RCAN1 dysregulation as well as the contribution of *Rcan1* triplication to DS-related impacts on periodicity of the circadian clock, photic entrainment of locomotor patterns, rest-activity profiles, and rhythmicity of activity.

## Materials and methods

### Animals

*Rcan1* knockout (KO) mice with wild-type (WT) littermates [59] and RCAN1-overexpressing transgenic (*RCAN1* TG) mice with non-transgenic (NTG) littermates [7] were generated and genotyped as previously described. *Dp(16)1Yey/+* (*Dp16*) mice were generated as described previously [58] and crossed with *Rcan1*^*(+/−)*^ mice [59] to obtain *Dp16* and WT littermates with *Dp16* mice that have *Rcan1* restored to two copies (*Dp16/Rcan1*^*2N*^). All mice in this study have been backcrossed > 10 generations with C57BL/6J mice to normalize the genetic background of the different mutant strains. WT mice from each respective cross are regularly used to maintain an isogenic background between *Rcan1* KO, *RCAN1* TG, and *Dp16* strains. All mice were bred in the same on-site facility with ambient temperature at 20–25°C and humidity 15–65%, weaned on post-natal day (PND) 21, and provided food (Envigo Teklad 2914 irradiated rodent diet; Harlan, Madison, WI) and water ad libitum. With the exception of free-running experiments conducted in constant darkness (DD), all mice were maintained on a standard 12:12 h light:dark cycle (LD12:12) with lights on at 07:00 as ZT0. Wheel running experiments with the *Rcan1* KO, *Rcan1* WT, *RCAN1* TG, and NTG mice utilized between-subjects designs wherein animals were assigned to either an LD12:12 or DD regimen and tested at either PND 90–180 (young) or PND 270–420 (aged). Wheel running experiments with the *Dp16*, *Dp16/Rcan1*^*2N*^, and WT mice utilized a within-subjects design with young (PND 90–180) mice wherein all animals were tested first in LD12:12 for 2 weeks and then transferred to DD for another 2 weeks of testing. Both sexes of each genotype were tested over multiple independent cohorts with litter-matched mice for all experiments. For each outcome measure, the sample sizes for each group are indicated on the corresponding bar plots within all figure panels. All housing and experimental conditions were approved by the Institutional Animal Care and Use Committee at the University of Colorado Boulder and conformed to the *Guide for the Care and Use of Laboratory Animals* (8th Ed.) from the National Institutes of Health.

### Wheel running data collection

For *Rcan1* KO, *Rcan1* WT, *RCAN1* TG, and NTG cohorts, home cage wheel running of singly housed mice maintained in either LD12:12 or DD was wirelessly recorded in 1-min intervals for a minimum of seven (LD12:12) or ten (DD) consecutive days (between-subjects design). For *Dp16*, *Dp16/Rcan1*^*2N*^, and WT cohorts, home cage wheel running of singly housed mice was wirelessly recorded for fourteen consecutive days in LD12:12 followed by fourteen consecutive days in DD (within-subjects design). Home cage wheel (Cat# ENV-047, Med Associates, St. Albans, VT) revolution data were collected using Running Wheel Manager Data Acquisition Software v1.06 (Cat# SOF-861, Med Associates, St. Albans, VT). The intensity of ambient lighting for light-entrained (LD12:12) wheel running experiments was 250 lux during the light phase and zero lux during the dark phase. Free-running (DD) experiments were conducted at constant zero lux with intermittent use of dim red lamps (<1 lux) to illuminate animal care tasks in the testing room. For all datasets, the first three (*Rcan1* KO, *Rcan1* WT, *RCAN1* TG, and NTG mice) or seven (*Dp16, Dp16/Rcan1*^*2N*^, and WT mice) 24-h intervals of raw wheel revolution data were excluded as the habituation period in order to mitigate the potential impacts of transients, aftereffects, acquisition time, and other experimental design-related and uncontrollable variables [60].

Notably, the between-subjects design utilized for LD12:12 and DD wheel running experiments in the *Rcan1* KO, *Rcan1* WT, RCAN1 TG, and NTG cohorts may confer potential confounds that warrant consideration when interpreting the data obtained therefrom. However, the substantial sample sizes comprising these datasets diminish the possible impacts of any confounds stemming from the between-subjects study design. Conversely, the within-subjects design utilized for LD12:12 and DD wheel running experiments in the *Dp16*, *Dp16/Rcan1*^*2N*^, and WT cohorts does not have such potential confounds. An additional limitation of the design of the present study is the differing exclusion windows utilized for processing of raw actigraphy data among *Rcan1* KO, *Rcan1* WT, *RCAN1* TG, and NTG mice relative to *Dp16*, *Dp16/Rcan1*^*2N*^, and WT mice. However, the wheel running phenotypes of *Dp16*, *Dp16/Rcan1*^*2N*^, and WT mice when a 3-day actigraphy data exclusion window is applied (data not shown) are comparable to those reported utilizing a 7-day exclusion window. These results support the validity of cross-model comparisons despite the differences in data exclusion windows utilized for actigraphic analyses of *Rcan1* KO, *Rcan1* WT, *RCAN1* TG, and NTG cohorts versus *Dp16*, *Dp16/Rcan1*^*2N*^, and WT cohorts.

### Periodic analyses

Analyses of the photic-entrained and circadian periodicity of wheel running were conducted as previously described [61]. Briefly, to identify the fundamental period and any discrete harmonics, minute-binned raw wheel revolution data for each animal were subjected to frequency decomposition via harmonic regression at Fourier frequencies using the following equation:

$$Y(t)={A}_j\sin \left(2\pi t/{\tau}_j\right)+e(t),j=24,12,8,6,4,3,2,1\dots$$

where *Y* = wheel revolutions, *t* = time, *A* = amplitude (*y*_max_ − *y*_mid_), *τ* = period (cycle duration, in hours), and *e*(*t*) = error term.

The zero-amplitude *F*-test was then applied to test the null hypothesis of zero amplitude for the fundamental period and any harmonics identified by frequency decomposition. For all (light-entrained and free-running) subjects, only the fundamental near-24-h period had significant non-zero amplitude. Fundamental period estimates were collapsed by genotype for statistical analysis.

### Rhythmometric analyses

Wheel running rhythms were analyzed as previously documented (Buck et al., 2019). Summarily, wheel running rhythms were parameterized by the MESOR (oscillatory mean), amplitude (oscillatory range), and acrophase (oscillatory phase, latency to peak activity). To estimate these parameters, the fundamental period of the wheel running rhythm for each subject was incorporated into a single-component cosinor regression model defined by the following formula:

$$Y(t)=M+A\cos \left(2\pi /\tau +\phi \right)+e(t)$$

where *Y* = wheel revolutions, *t* = time, *M* = MESOR (y_mid_), *A* = amplitude (*y*_max_ − *y*_mid_), *τ* = period (cycle duration, in hours), *ϕ* = acrophase (value of *t* at *y*_max_), and *e*(*t*) = error term.

This model was applied to minute-binned raw wheel revolution data, and goodness of model fit was verified by the Runs Test for each subject. The individual parameter of rhythm estimates obtained were collapsed by genotype and, where appropriate, age for subsequent statistical analysis.

### Western blot analysis

Hippocampal and suprachiasmatic nuclei (SCN)-enriched hypothalamic tissue were dissected from young WT, *Rcan1* KO, *Dp16*, and *Dp16*/*Rcan1*^***2N***^ mice (PND 90–180) maintained on an LD12:12 schedule but not provided access to running wheels to avoid potential confounds of voluntary exercise on RCAN1 expression. Tissue was collected from 5 to 8 mice of mixed sexes for each time point. Total protein extracts from the tissues were prepared for western blotting as described previously [7]. Briefly, tissues were homogenized by sonication in lysis buffer containing (in mM) 10 HEPES pH 7.4, 150 NaCl, 50 NaF, 1 EDTA, 1 EGTA, and 10 Na_4_P_2_O_7_ with 1X protease inhibitor cocktail III and 1X phosphatase inhibitor cocktails II and III (Sigma-Aldrich, St. Louis, MO). Twenty micrograms of protein were then prepared in Laemmli sample buffer, resolved on 4–12% Bis-Tris gradient gels, blotted on polyvinylidene difluoride membranes, and probed with RCAN1 (Cat# D6694, Sigma-Aldrich, St. Louis, MO), brain and muscle ARNT-like factor 1 (BMAL1; Cat# sc-365645, Santa Cruz Biotechnology, Dallas, TX), and β-tubulin (Cat# ab11308, Abcam, Cambridge, MA) antibodies using standard techniques. Primary antibodies were detected with horse radish peroxidase-conjugated secondary antibodies (Promega, Madison, WI). Blots were developed by application of Enhanced Chemiluminescence substrate (GE Healthcare Life Sciences), and immunoreactive signals were acquired and densitometrically quantified as previously described [7]. Optical density (OD) measurements were normalized by β-tubulin levels and presented relative to ZT11 levels.

### Mouse brain tissue immunostaining

*Rcan1* WT mice were perfused at ZT11, and the brains were fixed in 4% paraformaldehyde (PFA) for 24 h before being transferred to 30% sucrose in PBS for 24 h minimum at 4 °C for cryoprotection. Brains were then sectioned coronally at 30 μm on a cryostat (Leica). Fluorescent immunostaining was performed as described previously with minor changes [62]. Briefly, free-floating brain sections containing SCN were washed with PBS-T (1X PBS containing 0.5% Triton X-100) and blocked for 1 h at room temperature (RT) in staining buffer containing 0.05 M Tris pH 7.4, 0.9% NaCl, 0.25% gelatin, 0.5% Triton X-100, and 5% donkey serum. Slices were then incubated for 48 h at 4 °C with a combination of primary antibodies against RCAN1 (1:250, Sigma, D6694), BMAL1 (1:100, Santa Cruz, SC-365645), and NeuN (1:1000, Novus, NBP1-92693) diluted in staining buffer. Following primary antibody treatment, slices were washed in PBS-T and incubated at RT for 2 h with a combination of Hoechst dye (1:3000, ThermoFisher), Alexa Fluor 488-conjugated anti-mouse IgG1 (1:500, Invitrogen), Cy3-conjugated anti-rabbit (1:250, Jackson ImmunoResearch), and Alexa Fluor 647-conjugated anti-mouse IgG2b (1:500, Invitrogen) secondary antibodies in staining buffer without donkey serum. Following two washes in PBS-T and one wash in PBS, slices were mounted and coverslipped with Mowiol. Z-stacks through the entire thickness of the brain slices were imaged using the Nikon A1R confocal microscope with all microscope parameters held constant across slices from the same experiment. Images are representative of three independent samples for each staining.

### Statistical analyses

Prior to statistical analysis, all datasets were screened for outliers using the ROUT test (*Q* = 5%) and confirmed outliers were excluded from analysis where appropriate. A maximum of three outliers were excluded per group for each dataset. All wheel running data were initially analyzed by multifactorial ANOVA to determine if there were effects of sex as a biological variable. No main effects of or interactions with sex were detected for any outcome measure; therefore, data were collapsed by sex for subsequent analysis by mixed effects ANOVA. For the *Rcan1* KO and *RCAN1* TG cohorts, genotype (*Rcan1* KO, *Rcan1* WT, *RCAN1* TG, or NTG) and age (young or aged) served as between-subjects factors while corresponding outcome measures of wheel running phenotypes served as the within-subjects factor. For *Dp16*, *Dp16/Rcan1*^*2N*^, and WT mice, genotype served as the between-subjects factor and condition (LD12:12 or DD) and corresponding outcome measures of wheel running phenotypes served as within-subjects factors. Significant effects of and interactions among these factors were followed by Bonferroni’s multiple comparisons post hoc test. Analyses were conducted using R (https://cran.r-project.org) and SPSS 26 (IBM Analytics, Armonk, NY) and data were visualized using GraphPad Prism 8.1.1 (GraphPad Software, La Jolla, CA). For all analyses, the threshold for statistical significance (*α*) was set to 0.05 and adjusted for multiple comparisons.

For periodometric assessment of the *Rcan1* KO and *RCAN1* TG cohorts, the light-entrained and endogenous periodicity of wheel running were compared among young and aged *Rcan1* WT, *Rcan1* KO, NTG, and *RCAN1* TG mice by mixed ANOVA with genotype and age as between-subjects factors. For periodometric assessment of the *Dp16*, *Dp16/Rcan1*^*2N*^, and WT mice, genotype served as the between-subjects factor and condition (LD12:12 or DD) and outcome measure (light-entrained period length or endogenous period length) served as within-subjects factors.

For the assessment of light-entrained diurnal wheel running patterns and rhythms in the *Rcan1* KO, *Rcan1* WT, *RCAN1* TG, and NTG mice, measures of mean daily wheel revolutions during the light (inactive) phase (ZT0-ZT12) and dark (active) phase (ZT12-ZT24) were compared among groups by mixed ANOVA with genotype and age as between-subjects factors and outcome measure (light phase wheel running, dark phase wheel running, or percentage of total daily wheel running occurring in the light phase) as the within-subjects factor. Parameter of rhythm estimates were compared among the groups by mixed ANOVA with the between-subjects factors genotype and age and the within-subjects factor outcome measure (MESOR, amplitude, or acrophase).

For preparatory analyses of free-running wheel running datasets in all animals, daily activity onsets were identified by linear regression to enable quantification of wheel running during the rho (inactive) phase (occurring between the offset and onset of daily activity) and the alpha (active) phase (occurring between the onset and offset of daily activity). Time intervals for free-running experiments were represented in circadian units of time (circadian hours). To calculate the duration of circadian hours for each mouse, its endogenous period length (in conventional hours) was divided by twenty-four. The average time (in circadian hours) of daily activity onset was designated circadian time (CT) 12 for each animal. Prior to rhythmometric analysis of free-running mice, minute-binned raw wheel revolution data for each subject were aligned using CT12 as the reference point.

For assessment of free-running wheel running patterns and rhythms in the *Rcan1* KO, *Rcan1* WT, *RCAN1* TG, and NTG mice, mean daily wheel revolution data for the rho and alpha phases were compared among groups by mixed ANOVA with the between-subjects factor genotype and the within-subjects factor outcome measure (total wheel running, alpha phase wheel running, rho phase wheel running, or percentage of total daily wheel running occurring in the rho phase). Parameter of rhythm estimates were analyzed by mixed ANOVA with the between-subjects factor genotype and the within-subjects factor outcome measure (MESOR, amplitude, or acrophase).

For assessment of light-entrained and free-running wheel running patterns and rhythms in *Dp16*, *Dp16/Rcan1*^*2N*^, and WT mice, mean daily wheel revolution data for the inactive and active phases were analyzed by mixed ANOVA with the between-subjects factor genotype and the within-subjects factors condition (LD12:12 or DD) and outcome measure (total wheel running, active phase wheel running, inactive phase wheel running, or percentage of total daily wheel running occurring in the inactive phase). Parameter of rhythm estimates were analyzed by mixed ANOVA with the between-subjects factor genotype (*Dp16*, *Dp16/Rcan1*^*2N*^, and WT) and the within-subjects factors condition (LD12:12 or DD) and outcome measure (MESOR, amplitude, or acrophase).

For western blot analysis of RCAN1 and BMAL1 content in the hippocampus and SCN of WT mice, densitometric measurements were evaluated using independent samples *t*-test with the between-subjects factor ZT. For the *Rcan1* KO and *Dp16* cohorts, RCAN1 and BMAL1 content in the SCN was evaluated by two-way ANOVA with genotype (*Rcan1* KO and *Rcan1* WT or *Dp16*, *Dp16/Rcan1*^*2N*^, and WT) and ZT as between-subjects factors. Significant effects and interactions of these factors were followed by Tukey’s multiple comparisons post hoc test.

## Results

### RCAN1 mediates the circadian periodicity but not the photic entrainment of wheel running

To probe the role of RCAN1 in disruptions of circadian rhythms in DS, AD, and aging, we examined daily locomotor activity rhythms of wheel running behavior in two age groups of *Rcan1* KO mice with WT littermates and *RCAN1* TG mice with NTG littermates. In the young group, mice were tested at 3–6 months old, equivalent to early adulthood [63] before clinical AD onset. In the aged group, mice were tested at 9–14 months old, corresponding to middle age in humans [63] when aging-related dysfunction and preclinical AD symptoms emerge in the general population while in DS nearly all individuals have developed AD neuropathology [2, 64–66]. Given previous studies demonstrating that CaN [50, 52] and the *Drosophila* RCAN1 ortholog *sra* [49, 53] regulate the photic entrainment of circadian activity rhythms, we investigated whether RCAN1 similarly modulates light-entrained wheel running in mice. We also monitored free-running wheel activity of *Rcan1* KO and *RCAN1* TG mice in constant darkness (DD) to assess the integrity of their circadian clock and strength of their circadian rhythm.

**Fig. 1:**
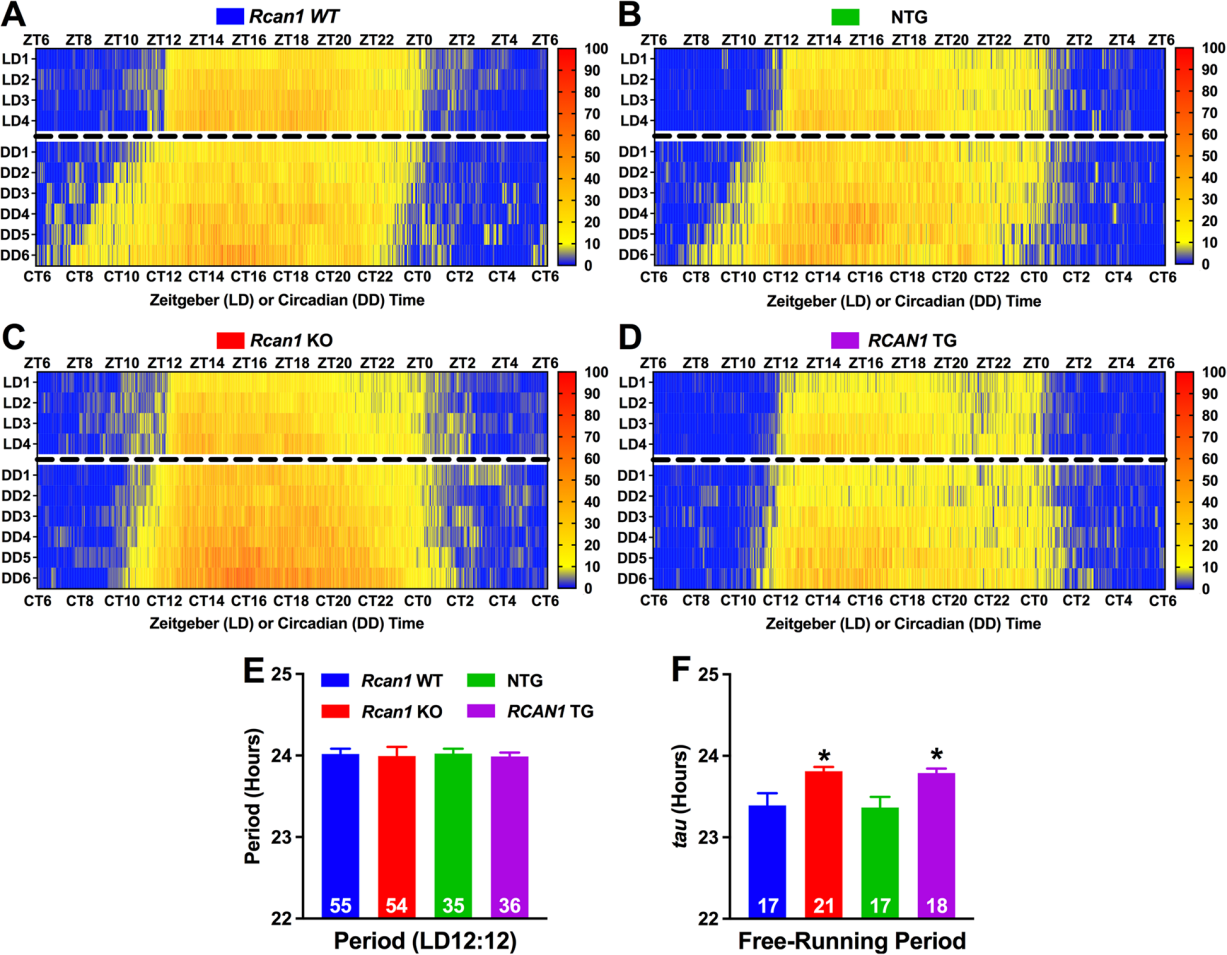
RCAN1 mediates the circadian periodicity but not the photic entrainment of wheel running. Heatmap-style actograms visualizing mean wheel revolution data for two distinct cohorts of mice tested in either LD12:12 conditions for a minimum of 7 days (days 4−7 displayed) or DD conditions for a minimum of 9 days (days 4−9 displayed) for **A** *Rcan1* WT, **B** *Rcan1* KO, **C** NTG, and **D** *RCAN1* TG groups. **E** Light-entrained diurnal period length of wheel running rhythms in LD12:12. There were no group differences in light-entrained period length. **F** Circadian (free-running) period length of wheel running rhythms in constant darkness. Young *Rcan1* KO and *RCAN1* TG mice have lengthened endogenous periods (tau) relative to young *Rcan1* WT and NTG mice, respectively. Light-entrained *N* = 55 *Rcan1* WT, 54 *Rcan1* KO, 35 NTG, 36 *RCAN1* TG mice; free-running *N* = 17 *Rcan1* WT, 21 *Rcan1* KO, 17 NTG, 18 *RCAN1* TG mice. All data are mean ± S.E.M. *p < 0.05

Mean actograms of wheel running behavior in *Rcan1* WT (Fig. 1A), *Rcan1* KO (Fig. 1B), NTG (Fig. 1C), and *RCAN1* TG (Fig. 1D) mice under LD12:12 or DD conditions revealed striking RCAN1-dependent differences in locomotor activity profiles. To quantify these differences, we first analyzed the daily periodicity of wheel running in LD12:12 or DD. We found a significant genotype × outcome measure interaction (*F*_6,307_ = 2.62; *p =* 0.017). No main effects of or interactions with age were detected, so group data were collapsed by age for further analysis. All genotypes showed a similar light-entrained period length (Fig. 1E), indicating that RCAN1 levels do not affect photic entrainment of circadian wheel running. By contrast, the endogenous period was lengthened in free-running *Rcan1* KO (*p =* 0.041) and *RCAN1* TG (*p =* 0.027) mice compared with *Rcan1* WT and NTG littermate controls, respectively (Fig. 1F). These data suggest that RCAN1 functions to modulate the periodicity of circadian locomotor activity rhythms.

### RCAN1 knockout and overexpression alter active and inactive phase wheel running patterns in light-entrained young but not aged mice

**Fig. 2:**
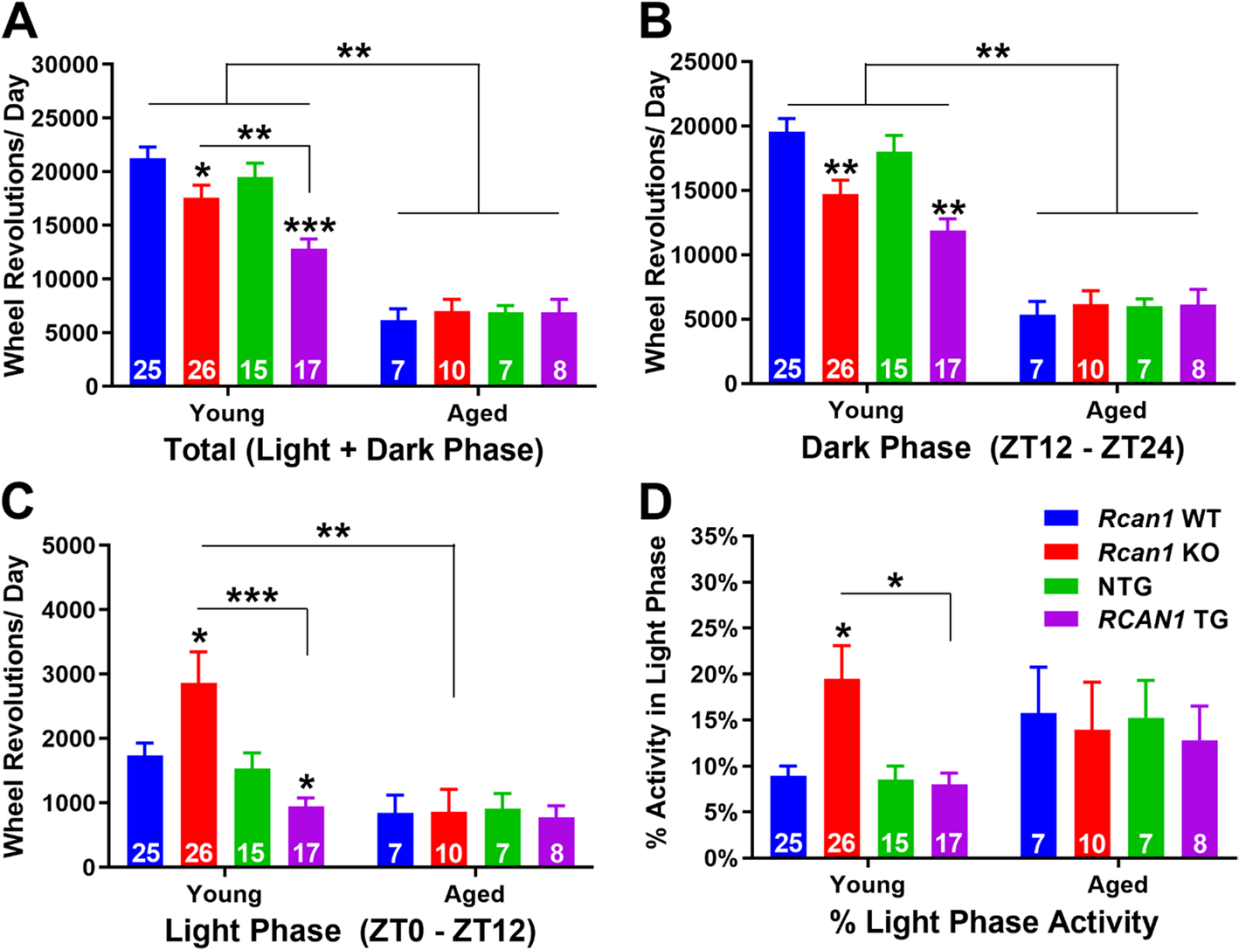
RCAN1 knockout and overexpression alter active and inactive phase wheel running patterns in light-entrained young but not aged mice. **A** Mean total daily wheel running of light-entrained young (left) and aged (right) mice. Young *Rcan1* KO and *RCAN1* TG mice exhibit reduced total daily wheel running relative to young *RCAN1* WT and NTG controls, respectively. Compared with young mice, aged mice showed decreased total daily wheel running. **B** Mean daily dark phase (ZT12-ZT24) wheel running of light-entrained young (left) and aged (right) mice. Young *Rcan1* KO and *RCAN1* TG mice are hypoactive during the dark phase compared with young *Rcan1* WT and NTG controls, respectively. Compared with young mice, aged mice showed decreased daily wheel running during the dark phase. **C** Mean daily light phase (ZT0-ZT12) wheel running of light-entrained young (left) and aged (right) mice. Young *Rcan1* KO mice are hyperactive during the light phase compared with both young *Rcan1* WT and *RCAN1* TG mice as well as with aged *Rcan1* KO mice. By contrast, young *RCAN1* TG mice are hypoactive compared with young NTG controls. **D** Mean percentage of total daily wheel running during the light phase for light-entrained young (left) and aged (right) mice. Young *Rcan1* KO mice have an increased percentage of total daily activity occurring during the light phase compared with young *Rcan1* WT and *RCAN1* TG mice. Young *N* = 25 *Rcan1* WT, 26 *Rcan1* KO*,* 15 NTG, 17 *RCAN1* TG mice; aged *N* = 7 *Rcan1* WT, 10 *Rcan1* KO, 7 NTG, 8 *RCAN1* TG mice. All data are mean ± S.E.M. *p < 0.05; **p < 0.01; ***p < 0.001

Considering the actigraphic alterations observed in *Rcan1* KO and *RCAN1* TG mice (Fig. 1A–D), we next analyzed the intensity of light-entrained total daily wheel running (Fig. 2A) as well as daily wheel running during the dark (active) (Fig. 2B) and light (inactive) (Fig. 2C) phases. To analyze the distribution of wheel running throughout an average day, we also determined the percentage of daily wheel running during the light phase (Fig. 2D). There were significant interactions of genotype × age (*F*_3,192_ = 3.22; *p=*0.027), age × outcome measure (*F*_2,192_=73.09; *p* < 1.0E−15), and genotype × age × outcome measure (*F*_6,192_ = 3.38; *p =* 0.017). In the young group, daily total wheel running was reduced both in *Rcan1* KO mice compared with *Rcan1* WT controls (*p =* 0.031) and in *RCAN1* TG mice compared with NTG controls (*p =* 3.0E−4) (Fig. 2A). Furthermore, *RCAN1* TG mice exhibited reduced total daily wheel running relative to *Rcan1* KO mice (*p =* 0.005; Fig. 2A). Across the dark and light phases, however, the wheel running patterns of *Rcan1* KO and *RCAN1* TG mice diverged. During the dark phase, both young *Rcan1* KO and *RCAN1* TG mice displayed lower daily wheel running compared with *Rcan1* WT (*p =* 0.002) and NTG (*p =* 0.003) controls, respectively (Fig. 2B), indicating active phase hypoactivity. During the light phase, on the other hand, young *Rcan1* KO mice were hyperactive compared with young *Rcan1* WT (*p =* 0.023) and *RCAN1* TG (*p =* 0.003) mice, whereas young *RCAN1* TG mice were hypoactive relative to NTG littermates (*p =* 0.031) (Fig. 2C). Additionally, young *Rcan1* KO mice exhibited an increased percentage of total daily activity during the light phase compared with young *Rcan1* WT (*p =* 0.011) and *RCAN1* TG (*p =* 0.017) mice (Fig. 2D). Together, these results indicate that total wheel running in an average day was reduced in both young *Rcan1* KO and *RCAN1* TG mice on the LD12:12 schedule, while young *Rcan1* KO mice alone shifted wheel running normally occurring in the dark phase to the light phase.

No differences in the intensity and distribution of light-entrained wheel running were detected among the aged groups. However, consistent with the known decline in locomotor activity with age [24], aged *Rcan1* WT and NTG mice showed reduced total daily wheel running compared with young *Rcan1* WT (*p =* 3.7E−15) and NTG (*p =* 1.2E−10) mice, respectively (Fig. 2A). Similarly, dark phase wheel running was decreased in aged *Rcan1* WT (*p =* 2.6E−8) and NTG (*p =* 5.0E−6) mice compared with their respective young counterparts (Fig. 2B). Young *Rcan1* KO and *RCAN1* TG mice both showed reduced daily total wheel running (Fig. 2A) and dark phase wheel running (Fig. 2B) in the direction of aged groups, implying that abnormal RCAN1 levels may facilitate premature aging-like phenotypes.

### RCAN1 knockout and overexpression similarly attenuate the light-entrained diurnal rhythmicity of wheel running in young but not aged mice

**Fig. 3:**
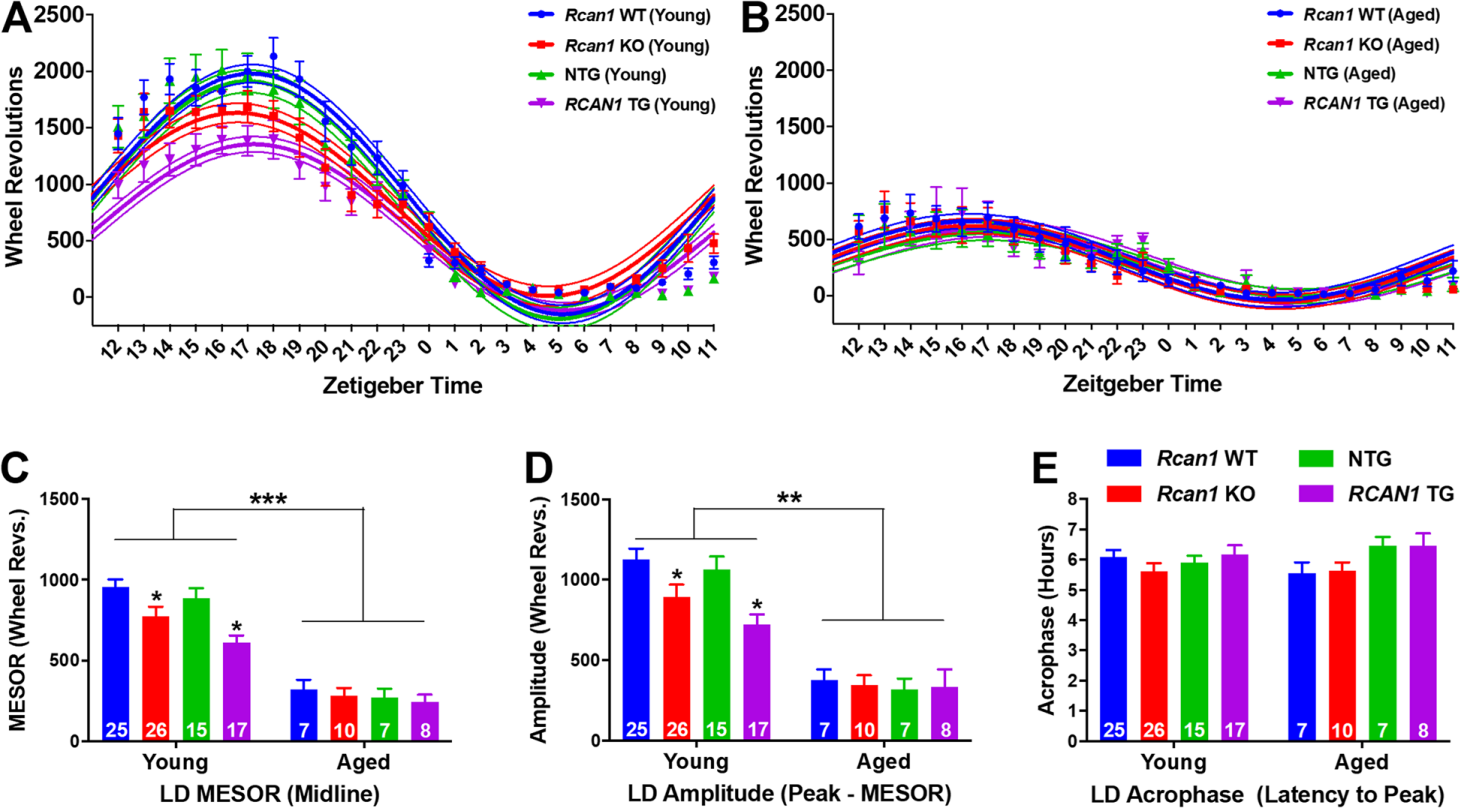
RCAN1 knockout and overexpression similarly attenuate the light-entrained diurnal rhythmicity of wheel running in young but not aged mice. Plots of average daily wheel revolutions collapsed into hourly bins (floating points depicting mean ± S.E.M) with superimposed single-harmonic regression curve fits (mean ± 95% CI bands) for **A** young and **B** aged mice. **C** Mean daily MESOR estimates for wheel running rhythms of light-entrained young (left) and aged (right) mice. Young *Rcan1* KO and *RCAN1* TG mice have decreased MESOR estimates versus young *Rcan1* WT and NTG mice, respectively. Aged *Rcan1* WT and NTG mice showed reduced MESOR estimates relative to young *Rcan1* WT and NTG mice, respectively. **D** Mean daily amplitude estimates for wheel running rhythms of light-entrained young (left) and aged (right) mice. Young *Rcan1* KO and *RCAN1* TG mice have reduced amplitude estimates compared with young *Rcan1* WT and NTG mice, respectively. Aged *Rcan1* WT and NTG mice showed decreased amplitude estimates relative to young *Rcan1* WT and NTG mice, respectively. **E** Mean daily acrophase estimates for wheel running rhythms of light-entrained young adult (left) and aged (right) mice. There were no group differences in acrophase estimates. Young *N* = 25 *Rcan1* WT, 26 *Rcan1* KO, 15 NTG, 17 *RCAN1* TG mice; aged *N* = 7 *Rcan1* WT, 10 *Rcan1* KO, 7 NTG, 8 *RCAN1* TG mice. All data are mean ± S.E.M. **p < 0.01; ***p < 0.001

Based on prior reports of reduced diurnal activity rhythm amplitudes in DS, AD, aging individuals, and animal models thereof [24, 31, 36, 56, 57], each of which exhibit RCAN1 upregulation, and in *sra* KO flies, which lack the *Drosophila* homolog of RCAN1 [49], we hypothesized that RCAN1 also regulates the rhythmicity of wheel running. Therefore, we examined the impact of RCAN1 knockout and overexpression on rhythmic characteristics of light-entrained wheel running with age. To estimate parameters of rhythm, cosinor analysis was used to curve-fit daily wheel running of young (Fig. 3A) and aged (Fig. 3B) *Rcan1* KO and *RCAN1* TG mice in LD12:12. The oscillatory mean (MESOR; Fig. 3C), range (amplitude; Fig. 3D), and phase (acrophase; Fig. 3E) of the fitted curves were estimated as measures of wheel running rhythmicity.

There were significant interactions of genotype × outcome measure (*F*_6,192_ = 2.46; *p =* 0.032), age × outcome measure (*F*_2,192_ = 64.38; *p =* 1.0E^−15^), and genotype × age × outcome measure (*F*_6,192_ = 3.60; *p =* 0.016) in light-entrained circadian rhythms of wheel running. Compared with their corresponding littermate controls, young *Rcan1* KO and *RCAN1* TG mice displayed reduced MESOR (*p =* 0.026 and *p =* 0.010, respectively; Fig. 3C) and amplitude (*p =* 0.047 and *p =* 0.028, respectively; Fig. 3D) estimates, indicating flattened circadian rhythmicity of wheel running. There were no differences among the young groups for acrophase estimates (Fig. 3E), suggesting RCAN1 does not play a role in the phasing of peak daily wheel running. No parameters of rhythm differed among aged groups (Fig. 3C–E), indicating that RCAN1 depletion and overexpression attenuate the strength of circadian locomotor rhythms in young, but not aged, mice. However, as expected, aged mice displayed dampened wheel running rhythmicity relative to young mice, indicated by the decreased MESOR and amplitude estimates in aged versus young *Rcan1* WT (*p =* 4.9E−11 and *p =* 7.5E−7, respectively) and aged versus young NTG (*p =* 1.1E−6 and *p =* 0.004, respectively) mice. Since both MESOR and amplitude estimates were reduced in young *Rcan1* KO and *RCAN1* TG mice toward aged levels, these data further suggest that abnormal RCAN1 levels may accelerate senescence phenotypes.

### RCAN1 knockout and overexpression bidirectionally perturb wheel running patterns in free-running young mice

**Fig. 4:**
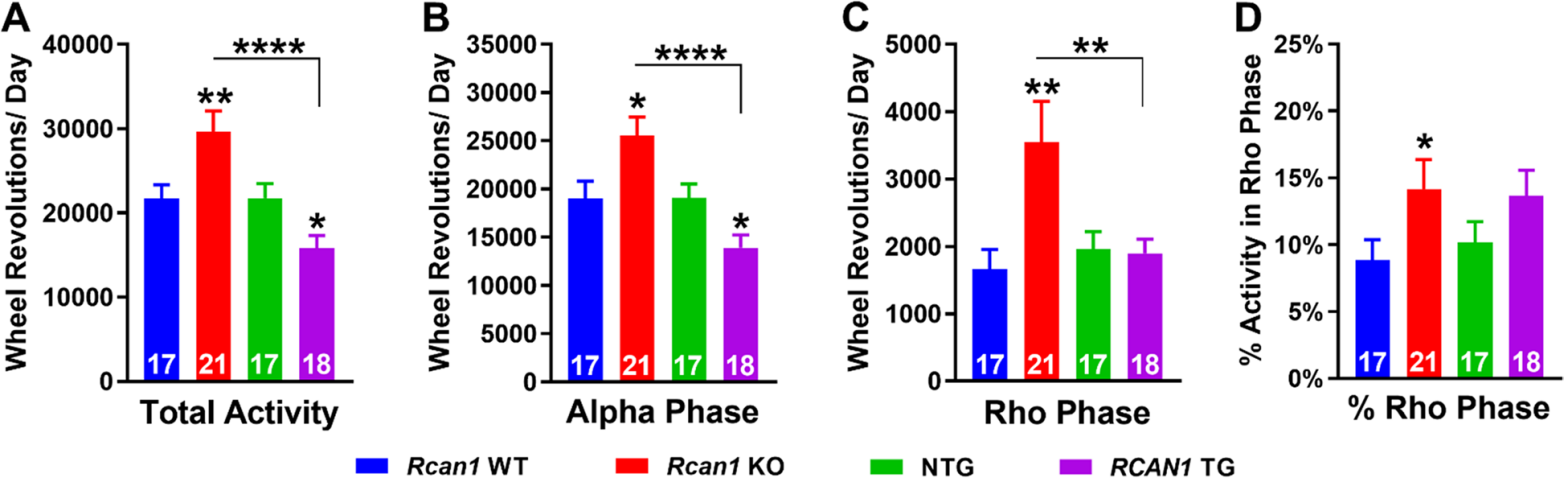
RCAN1 knockout and overexpression bidirectionally perturb wheel running patterns of free-running young mice. **A** Mean total (CT0-CT24) daily wheel running of free-running young mice. *Rcan1* KO mice exhibit increased total daily wheel running compared with *Rcan1* WT and *RCAN1* TG mice, while *RCAN1* TG mice displayed decreased total daily wheel running compared with NTG mice. **B** Mean daily alpha (active) phase wheel running of free-running young mice in DD*. Rcan1* KO mice show increased daily wheel running in the alpha phase compared with *Rcan1* WT and *RCAN1* TG mice, while *RCAN1* TG mice show decreased daily wheel running in the alpha phase relative to NTG mice. **C** Mean daily rho (inactive) phase wheel running of free-running young mice in DD*. Rcan1* KO mice exhibit elevated daily wheel running during the rho phase compared with *Rcan1* WT and *RCAN1* TG mice. **D** Mean percentage of total daily wheel running during the rho phase for free-running young mice. Rcan1 KO mice display an increased percentage of total daily wheel running in the rho phase compared with *Rcan1* WT mice, while *RCAN1* TG mice trended toward an increased percentage of total daily activity in the rho phase compared to NTG mice. *N* = 17 *Rcan1* WT, 21 *Rcan1* KO, 17 NTG, 18 *RCAN1* TG mice. All data are mean ± S.E.M. *p < 0.05; **p < 0.01; ****p < 0.0001

We also assessed wheel running profiles in the absence of light entrainment using DD (free-running) conditions. Given the altered free-running locomotor activity reported in RCAN1-overexpressing DS models [55, 57] and *sra* KO flies [49, 53], we posited that RCAN1 additionally modulates circadian wheel running rhythms. In support of this theory, we found RCAN1-dependent effects on the circadian period of wheel running rhythms (Fig. 1F). To determine if RCAN1 knockout and overexpression also affect the intensity and distribution of daily free-running wheel activity, we next examined total wheel running (Fig. 4A), wheel running during the alpha (active) (Fig. 4B) and rho (inactive) (Fig. 4C) phases, and the percentage of total daily wheel running during the rho phase (Fig. 4D) in free-running young *Rcan1* KO and *RCAN1* TG mice.

There was a significant genotype × outcome measure interaction (*F*_9,174_ = 8.14; *p =* 4.8E^−9^) for daily wheel running profiles among free-running mice. Relative to *Rcan1* WT and *RCAN1* TG mice, *Rcan1* KO mice exhibited higher total daily free-running wheel activity (*p =* 0.008 and *p =* 5.0E−6, respectively; Fig. 4A), resulting from increased wheel running during both the alpha (*p =* 0.048 and *p =* 4.0E−5, respectively; Fig. 4B) and rho (*p =* 0.003 and *p =* 0.009, respectively; Fig. 4C) phases. *Rcan1* KO mice also exhibited an increased percentage of total daily wheel running in the rho phase (*p =* 0.047) compared with *Rcan1* WT controls (Fig. 4D), indicating a shift toward an increased proportion of total daily wheel running during the inactive phase. By contrast, total daily wheel activity of free-running *RCAN1* TG mice was decreased (*p =* 0.030, Fig. 4A), largely stemming from decreased wheel running during the alpha phase (*p =* 0.039; Fig. 4B) compared with NTG controls. Therefore, abolition of RCAN1 led to hyperactivity whereas upregulation of RCAN1 led to hypoactivity in free-running mice, suggesting that RCAN1 levels titrate circadian locomotor activity patterns in the absence of light entrainment. *RCAN1* TG mice displayed no difference in rho phase activity versus NTG controls (Fig. 4C) but trended (*p =* 0.17) toward an increased percentage of total daily wheel running in the rho phase (Fig. 4D) like *Rcan1* KO mice. Together, these results demonstrate that RCAN1 knockout and overexpression disrupt free-running rest-activity profiles.

### RCAN1 knockout and overexpression elicit divergent alterations in the circadian rhythmicity of wheel running in young mice

**Fig. 5:**
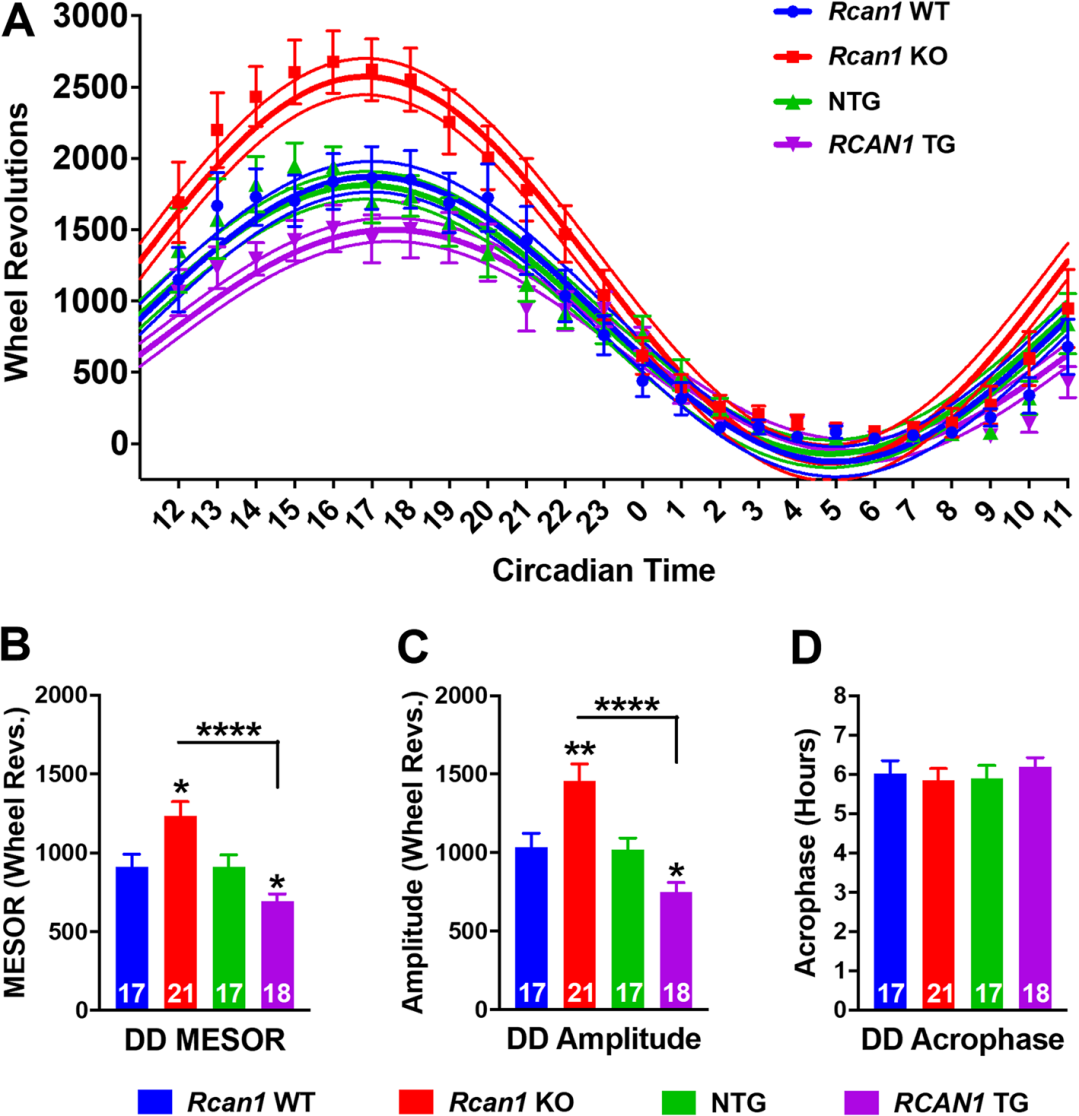
RCAN1 knockout and overexpression elicit divergent alterations in the circadian rhythmicity of wheel running in young mice. **A** Plot of average daily wheel revolutions collapsed into hourly bins (floating points depicting mean ± S.E.M) with superimposed single-harmonic regression curve fits (mean ± 95% CI bands) for free-running young mice. **B** Mean daily MESOR estimates for wheel running rhythms of free-running young mice. *Rcan1* KO mice exhibit increased daily MESOR estimates compared with *Rcan1* WT and *RCAN1* TG mice, while *RCAN1* TG mice display decreased daily MESOR estimates relative to NTG mice. **C** Mean daily amplitude estimates for wheel running rhythms of free-running young mice. *Rcan1* KO mice exhibit increased daily amplitude estimates compared with *Rcan1* WT and *RCAN1* TG mice, while *RCAN1* TG mice display decreased daily amplitude estimates compared with NTG mice. **D** Mean daily acrophase estimates for wheel running rhythms of free-running young mice. There were no group differences in acrophase estimates. *N* = 17 *Rcan1* WT, 21 *Rcan1* KO, 17 NTG, 18 *RCAN1* TG mice. All data are mean ± S.E.M. *p < 0.05; **p < 0.01; ****p < 0.0001

Considering the opposing consequences of RCAN1 deficiency and overexpression on the daily wheel running patterns of free-running young mice (Fig. 4) coupled with the RCAN1-mediated effects on diurnal rhythmicity of wheel running in light-entrained mice (Fig. 3), we postulated that RCAN1 may bidirectionally regulate the circadian rhythmicity of wheel running in young mice. To test this idea, we performed rhythmometric analysis of the wheel activity of free-running young *Rcan1* KO and *RCAN1* TG mice (Fig. 5A) to estimate the MESOR (Fig. 5B), amplitude (Fig. 5C), and acrophase (Fig. 5D) of their circadian wheel running rhythms. There was a significant genotype × outcome measure interaction (*F*_6,131_ = 8.52; *p =* 1.0E−7). Relative to *Rcan1* WT and *RCAN1* TG mice, *Rcan1* KO mice exhibited increased MESOR (*p =* 0.008 and *p =* 2.0E−5, respectively; Fig. 5B) and amplitude (*p =* 0.004 and *p =* 1.0E−6, respectively; Fig. 5C) estimates, indicating increased oscillatory means and intra-daily variability of endogenous circadian wheel running rhythms. By contrast, *RCAN1* TG mice displayed decreased MESOR (*p =* 0.043; Fig. 5B) and amplitude (*p =* 0.039; Fig. 5C) estimates compared with NTG controls, indicating that RCAN1 overexpression dampens the endogenous rhythmicity of daily wheel running. There were no differences in acrophase estimates among any groups (Fig. 5D), suggesting that RCAN1 levels regulate the strength and variability but not the phasing of endogenous locomotor activity rhythms. These data provide further evidence of disrupted circadian activity rhythms in young *Rcan1* KO and *RCAN1* TG mice.

### Circadian periodicity and photic entrainment of wheel running are unaltered in young *Dp16* mice

Given our findings of altered wheel running in young RCAN1-overexpressing mice and that *RCAN1* is triplicated in DS, we next characterized wheel running phenotypes in the *Dp16* mouse model for DS, which carries three *Rcan1* copies. To examine the specific role of RCAN1 in the DS mouse model, we also generated *Dp16* mice with *Rcan1* restored to two copies (*Dp16/Rcan1*^*2N*^) and assessed whether any wheel running alterations that *Dp16* mice exhibit could be normalized by *Rcan1* dosage correction. Because we observed that RCAN1 knockout and overexpression impacted wheel running in young mice, we compared the light-entrained (diurnal) and free-running (circadian) periodicity, patterning, and rhythmicity of wheel running among young WT, *Dp16* and *Dp16/Rcan1*^*2N*^ littermates. The mice underwent testing in LD12:12 conditions for 2 weeks, followed by constant darkness (DD) for two more weeks.

**Fig. 6:**
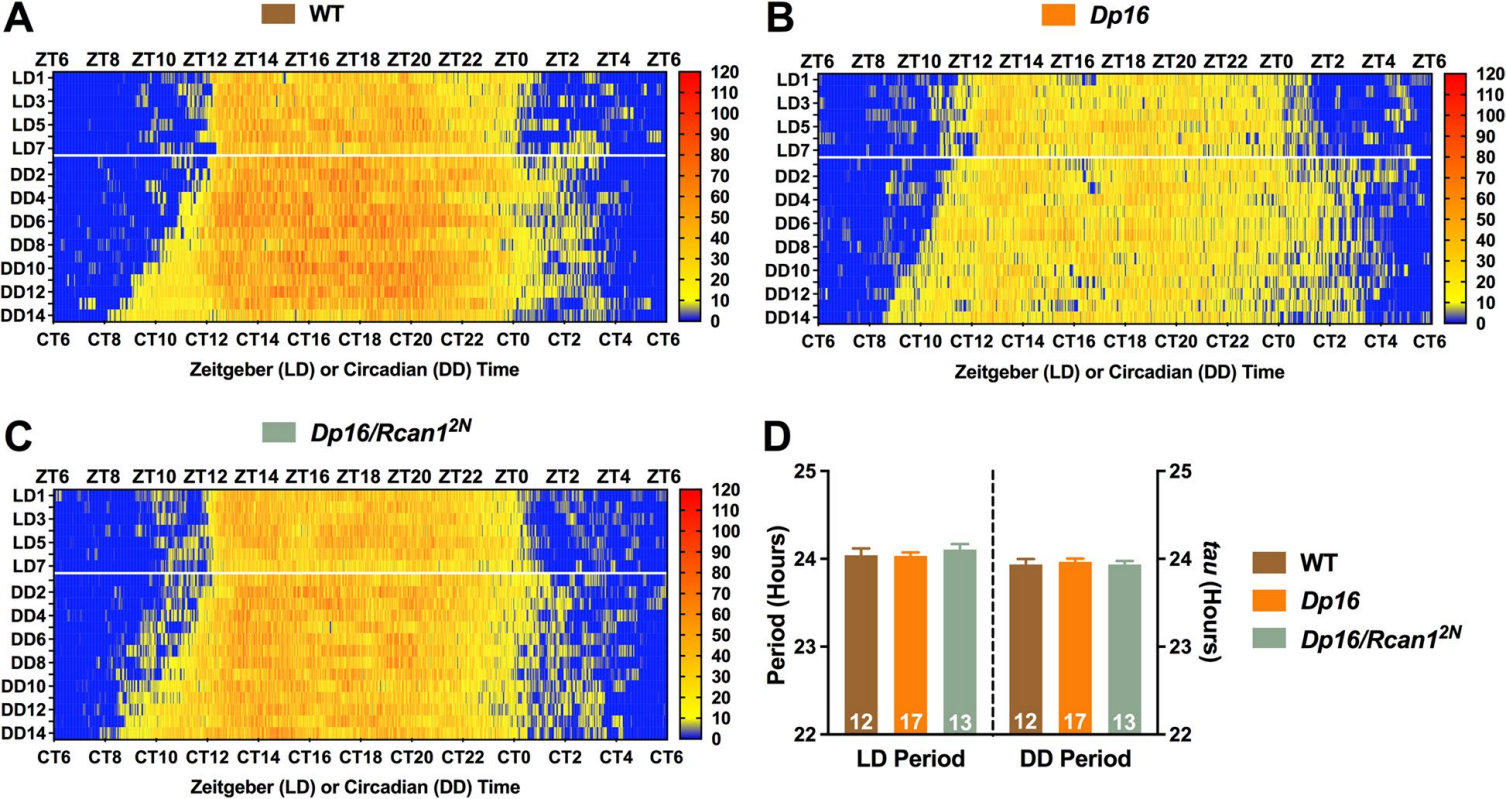
Photic entrainment and circadian periodicity of wheel running are unaltered in young *Dp16* mice independent of *Rcan1* copy number. Heatmap-style actograms visualizing mean wheel revolution data for **A** WT, **B** *Dp16*, and **C** *Dp16/Rcan1*^*2N*^ mice tested in LD12:12 conditions for 14 days (days 8–14 displayed) and immediately transferred to and tested in DD conditions for 14 days (days 1–14 displayed). **D** Mean wheel running period lengths for light-entrained (left) and free-running (right) young mice*.* No group differences in either light-entrained or free-running (endogenous) period length were detected. *N* = 12 WT, 17 *Dp16*, 13 *Dp16/Rcan1*^*2N*^ mice. All data are mean ± S.E.M.

Mean actograms of wheel running behavior revealed clear distinctions in locomotor activity profiles for WT (Fig. 6A), *Dp16* (Fig. 6B), and *Dp16/Rcan1*^*2N*^ (Fig. 6C) mice across LD12:12 and DD conditions. However, no main effects of or interactions among genotype, condition, or outcome measure were detected for the periodicity of wheel running. Both light-entrained diurnal and circadian period lengths (Fig. 6D) were comparable across groups. These data show that an extra copy of mouse homologs to HSA21 genes and correcting *Rcan1* dosage do not affect the periodicity of light-entrained diurnal or circadian locomotor activity rhythms in young *Dp16* mice.

### Altered wheel running patterns in light-entrained and free-running young *Dp16* mice are partially rescued by restoring *Rcan1* to disomic levels

**Fig. 7:**
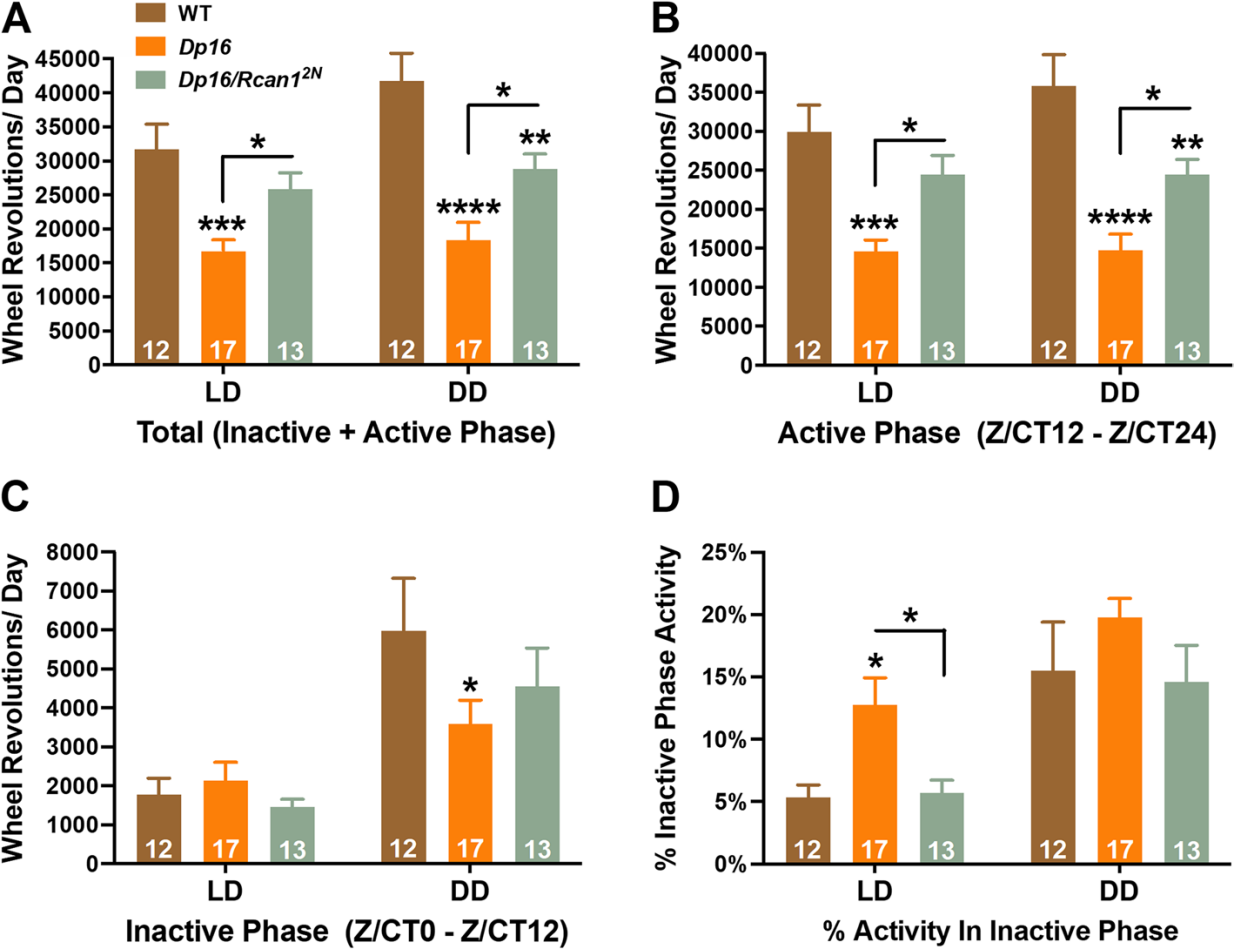
Altered wheel running patterns in light-entrained and free-running young *Dp16* mice are partially normalized by restoration of *Rcan1* to two copies. **A** Mean total daily wheel running of light-entrained (left) and free-running (right) young mice. Light-entrained young *Dp16* mice exhibited decreased total daily wheel running compared with WT and *Dp16/Rcan1*^*2N*^ mice. Free-running young *Dp16* mice displayed reduced total daily wheel running relative to both WT and *Dp16/Rcan1*^*2N*^ mice, and free-running young *Dp16/Rcan1*^*2N*^ mice exhibited decreased total daily wheel running versus WT mice. **B** Mean daily active phase wheel running of light-entrained (left) and free-running (right) young mice. Light-entrained young *Dp16* mice exhibited decreased active phase wheel running compared with WT and *Dp16/Rcan1*^*2N*^ mice. Free-running young *Dp16* mice displayed reduced active phase wheel running relative to both WT and *Dp16/Rcan1*^*2N*^ mice, and free-running young *Dp16/Rcan1*^*2N*^ mice exhibited decreased active phase wheel running versus WT mice. **C** Mean daily inactive phase wheel running of light-entrained (left) and free-running (right) young mice. No group differences were detected for inactive phase wheel running among light-entrained mice. However, free-running young *Dp16* mice displayed reduced inactive phase wheel running relative to WT mice. **D** Mean percentage of total daily wheel running during the inactive phase for light-entrained (left) and free-running (right) young mice. Light-entrained young *Dp16* mice exhibited increased percent inactive phase wheel running compared with WT and *Dp16/Rcan1*^*2N*^ mice. No group differences were detected for percent inactive phase wheel running among free-running mice. *N* = 12 WT, 17 *Dp16*, 13 *Dp16/Rcan1*^*2N*^ mice. All data are mean ± S.E.M. *p < 0.05; **p < 0.01; ***p < 0.001; ****p < 0.0001

We next measured the intensity of total daily wheel running in young *Dp16* mice under light-entrained or free-running conditions (Fig. 7A). To analyze the distribution of wheel running throughout an average day in LD12:12 or DD, we also measured daily wheel running of young *Dp16* mice during the active (Fig. 7B) and inactive (Fig. 7C) phases separately and determined the percentage of daily wheel running occurring in the inactive phase (Fig. 7D). In contrast to the periodicity, we found interaction effects of genotype × condition (*F*_2,37_ = 3.5; *p =* 0.042), genotype × outcome measure (*F*_6,72_ = 4.4; *p =* 0.001), and condition × outcome measure (*F*_3,37_ = 12.8; *p =* 7.0E−6) on daily wheel running patterns.

Post hoc testing revealed reduced total daily wheel running in *Dp16* mice under both LD12:12 (*p =* 5.0E−4) and DD (*p =* 1.0E−7) conditions compared with WT mice (Fig. 7A). Critically, total daily wheel running under LD12:12 also was reduced in *Dp16* mice compared with *Dp16/Rcan1*^*2N*^ mice (*p =* 0.044) while no significant difference was observed between *Dp16/Rcan1*^*2N*^ and WT mice (Fig. 7A). Under DD, *Dp16/Rcan1*^*2N*^ mice displayed total daily wheel running that was lower than in WT mice (*p =* 0.005) but greater than in *Dp16* mice (*p =* 0.018) (Fig. 7A). These results suggest that *Rcan1* dosage correction restores total wheel running in young *Dp16* mice at least partially to WT levels.

Similarly, during the active phases under both LD12:12 and DD conditions, *Dp16* mice showed less wheel running than WT (*p =* 1.0E−4 and *p =* 2.0E−7, respectively) and *Dp16/Rcan1*^*2N*^ (*p =* 0.014 and *p =* 0.015, respectively) mice (Fig. 7B). Compared with WT mice, active phase wheel running in *Dp16/Rcan1*^*2N*^ mice was not significantly different under LD12:12 but was reduced under DD conditions (*p =* 0.009) (Fig. 7B). These data indicate active phase hypoactivity in both light-entrained and free-running young *Dp16* mice that are mostly rescued by restoring *Rcan1* to disomic levels. No wheel running differences were detected during the inactive phase among light-entrained groups (Fig. 7C). However, during the inactive phase under DD, *Dp16* mice again showed decreased wheel running relative to WT mice (*p =* 0.047) while no differences were detected between *Dp16/Rcan1*^*2N*^ and *Dp16* or WT mice (Fig. 7C). When we examined the percentage of total daily wheel running in the inactive phase, *Dp16* mice showed an increase compared with WT (*p =* 0.041) and *Dp16/Rcan1*^*2N*^ (*p =* 0.047) mice under LD12:12 but no group differences were detected under DD conditions (Fig. 7D). Together, these results indicate that diurnal and circadian wheel running are reduced in *Dp16* mice, with more prominent effects during the active phase. These phenotypes were also largely dependent on *Rcan1* dosage because restoring *Rcan1* to two copies in *Dp16* mice reduced or eliminated them.

### Light-entrained diurnal and circadian wheel running rhythms are diminished in young *Dp16* mice and are partially normalized by restoring *Rcan1* to two copies

**Fig. 8:**
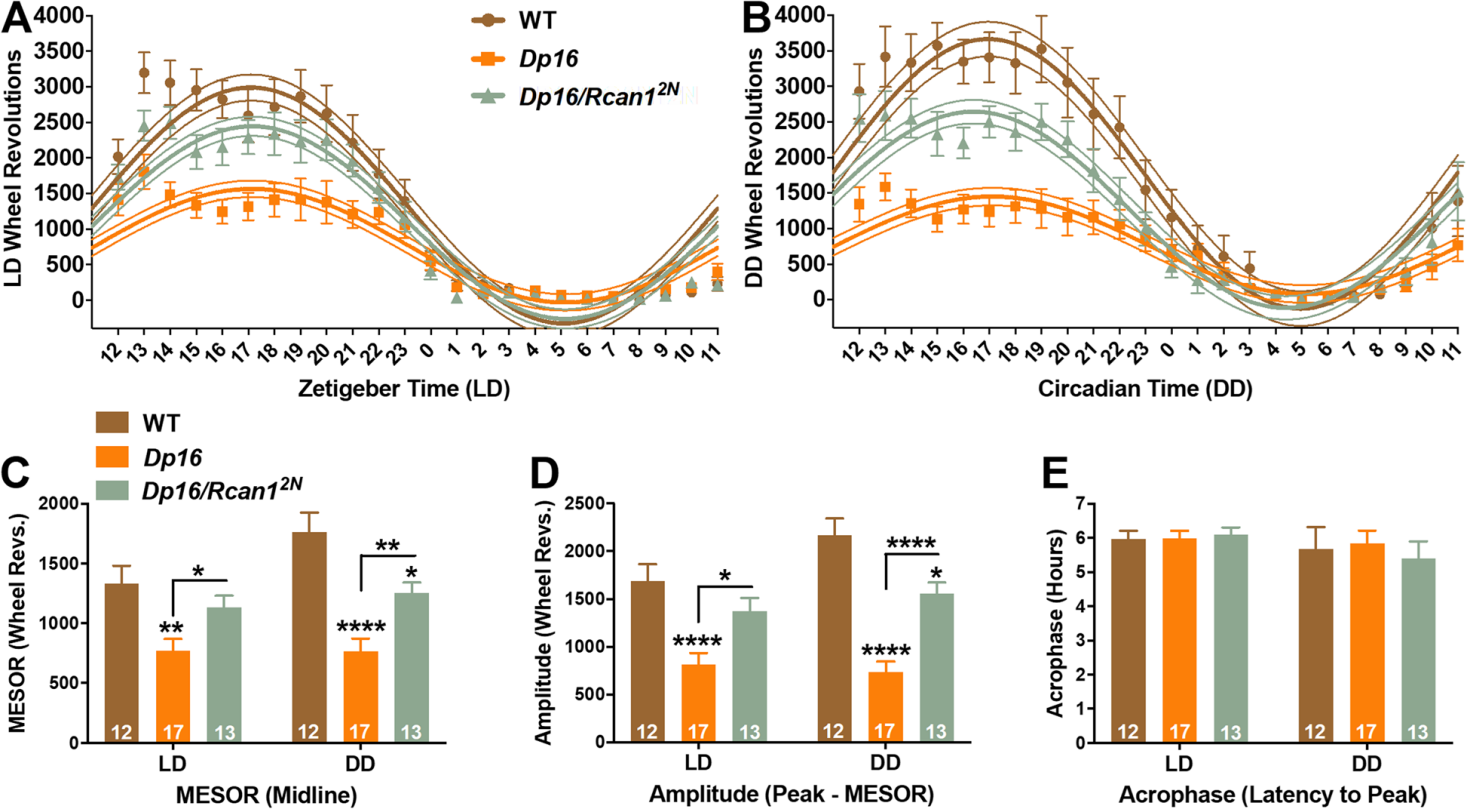
Light-entrained diurnal and circadian wheel running rhythms are diminished in young *Dp16* mice and are partially normalized by restoration of *Rcan1* to two copies. Plots of average daily wheel revolutions collapsed into hourly bins (floating points depicting mean ± S.E.M) with superimposed single-harmonic regression curve fits (mean ± 95% CI bands) for **A** light-entrained and **B** free-running young mice. **C** Mean daily MESOR estimates for wheel running rhythms of light-entrained (left) and free-running (right) young mice*.* Light-entrained young *Dp16* mice exhibited decreased MESOR estimates compared with WT and *Dp16/Rcan1*^*2N*^ mice. Free-running young *Dp16* mice displayed reduced MESOR estimates relative to both WT and *Dp16/Rcan1*^*2N*^ mice, and free-running young *Dp16/Rcan1*^*2N*^ mice exhibited decreased MESOR estimates versus WT mice. **D** Mean daily amplitude estimates for wheel running rhythms of light-entrained (left) and free-running (right) young mice. Light-entrained young *Dp16* mice exhibited decreased amplitude estimates compared to WT and *Dp16/Rcan1*^*2N*^ mice. Free-running young *Dp16* mice displayed reduced amplitude estimates relative to both WT and *Dp16/Rcan1*^*2N*^ mice, and free-running young *Dp16/Rcan1*^*2N*^ mice exhibited decreased amplitude estimates versus WT mice. **E** Mean daily acrophase estimates for wheel running rhythms of light-entrained (left) and free-running (right) young mice. There were no group differences in acrophase estimates. *N* = 12 WT, 17 *Dp16*, 13 *Dp16/Rcan1*^*2N*^ mice. All data are mean ± S.E.M. *p < 0.05; **p < 0.01; ****p < 0.0001

Based on our *RCAN1* TG results (Figs. 3 and 5), we hypothesized that *Rcan1* triplication in young *Dp16* mice may also impact wheel running rhythms. Therefore, we compared the characteristics of light-entrained diurnal and circadian wheel running rhythms in young WT, *Dp16*, and *Dp16/Rcan1*^*2N*^ mice. To this end, we used cosinor analysis as performed earlier to curve-fit their daily wheel running under LD12:12 (Fig. 8A) and DD (Fig. 8B) conditions. The oscillatory mean (MESOR; Fig. 8C), range (amplitude; Fig. 8D), and phase (acrophase; Fig. 8E) of the fitted curves were estimated as measures of wheel running rhythmicity. Significant group × condition (*F*_2,37_ = 5.0; *p=*0.012), group × outcome measure (*F*_4,72_ = 8.9; *p =* 7.0E−5), and condition × outcome measure (*F*_2,37_ = 4.7; *p =* 0.016) interactions were detected for daily wheel running rhythms.

Under both light entrainment and free-running conditions, *Dp16* mice exhibited decreased MESOR (*p =* 0.003 and *p =* 1.0E−7, respectively; Fig. 8C) and amplitude (*p =* 7.0E−5 and *p =* 2.0E−10, respectively; Fig. 8D) estimates compared with WT controls. These results indicate dampened wheel running rhythmicity in young *Dp16* mice similar to young *RCAN1* TG mice (Figs. 3 and 5). Consistent with the idea that RCAN1 levels regulate the oscillatory strength and range of entrained diurnal and circadian wheel running rhythms, *Dp16/Rcan1*^*2N*^ mice compared with *Dp16* littermates under both LD12:12 and DD exhibited increased MESOR (*p =* 0.047 and *p =* 0.008, respectively; Fig. 8C) and amplitude (*p =* 0.011 and *p =* 9.0E−5, respectively; Fig. 8D) estimates. Compared to WT littermates, no MESOR or amplitude differences were detected in *Dp16/Rcan1*^*2N*^ mice under LD12:12 (Fig. 8C, D), suggesting that *Rcan1* dosage correction in *Dp16* mice normalized these rhythmic characteristics. Under DD, *Dp16/Rcan1*^*2N*^ mice exhibited decreased MESOR (*p =* 0.013) and amplitude (*p =* 0.011) estimates compared with WT mice but not as severely as *Dp16* mice (Fig. 8C, D), suggesting that *Rcan1* dosage correction improved these rhythmic deficits in *Dp16* mice. As observed with young *RCAN1* TG mice (Figs. 3E and 5D), there were no differences in acrophase estimates among the groups (Fig. 8E), further suggesting that RCAN1 does not play a role in the phasing of entrained diurnal or circadian wheel running rhythms. Because MESOR and amplitude estimates were reduced in light-entrained and free-running *Dp16* mice and restoring *Rcan1* to two copies in *Dp16* mice suppressed these effects, these data provide additional support that RCAN1 overexpression disrupts diurnal and circadian activity rhythms.

**Fig. 9:**
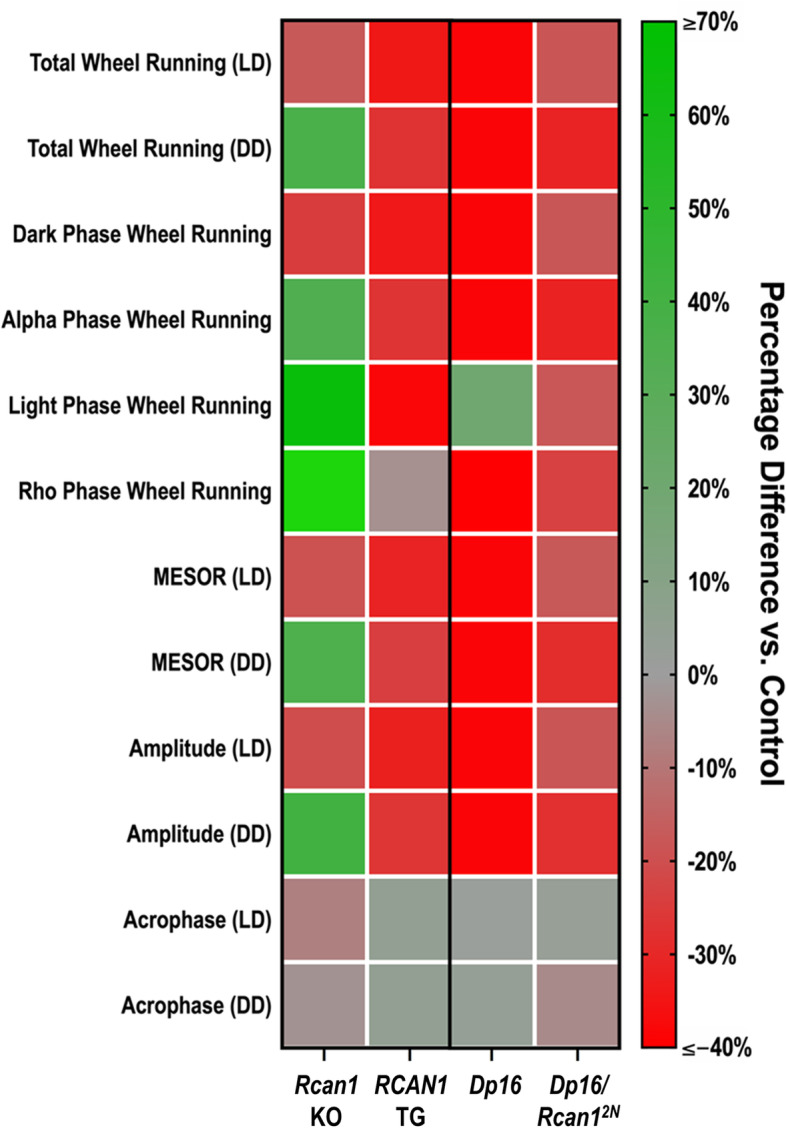
Survey of wheel running phenotypes in young *Rcan1* KO, *RCAN1* TG, *Dp16*, and *Rcan1* dosage-corrected *Dp16* mice. Dual-gradient heatmap depicting the percentage differences of young *Rcan1* KO and *RCAN1* TG mice relative to young *Rcan1* WT and NTG control mice, respectively (left horizontal axis) as well as young *Dp16* and *Dp16/Rcan1*^*2N*^ mice relative to young WT control mice (right horizontal axis) for measures of wheel running patterns and rhythms in light-entrained (LD) and free-running (DD) conditions (vertical axis). A percentage difference of zero indicates no difference (depicted in gray) relative to the corresponding control group, whereas percentage differences of ≤−40% and ≥70% indicate a 40% or greater decrease (depicted in red) and a 70% or greater increase (depicted in green), respectively, relative to the corresponding control group

Using a heat map, we summarized the wheel running phenotypes evinced among the young cohorts of *Rcan1* KO, *RCAN1* TG, *Dp16*, and *Dp16/Rcan1*^*2N*^ mice (Fig. 9). This overview highlights the bidirectional influences of RCAN1 levels on wheel running phenotypes. Additionally, it intimates that restoration of RCAN1 to disomic expression levels in *Dp16/Rcan1*^*2N*^ mice partially normalizes aberrant wheel running phenotypes conferred by the *Dp16* genotype.

### RCAN1 expression is normally arrhythmic in young mice

**Fig. 10:**
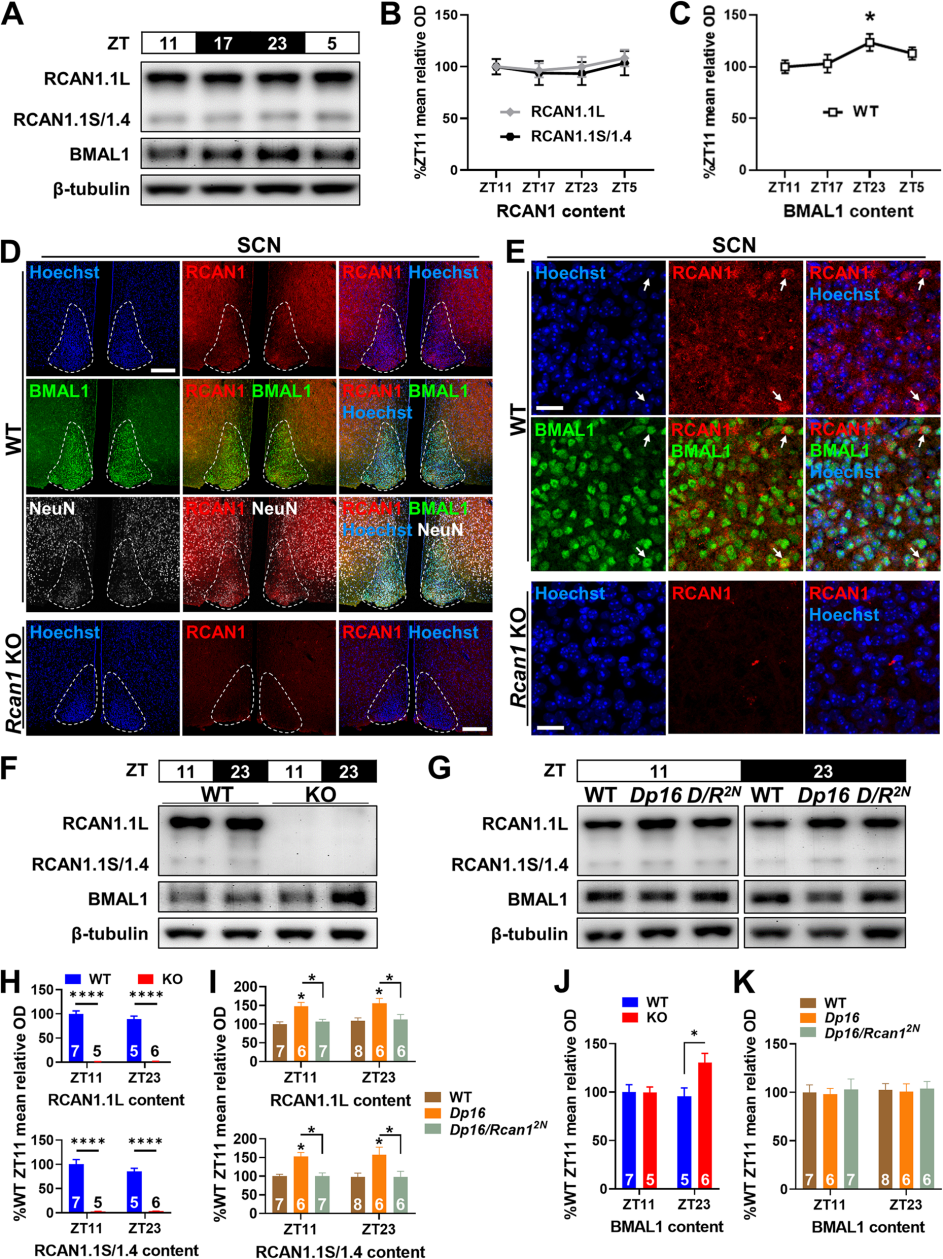
RCAN1 expression is normally arrhythmic in young mice. **A** Representative western blot images of RCAN1 and BMAL1 in the hippocampi of *Rcan1* WT adult mice (3–6 months old) at ZT11, ZT17, ZT23, and ZT5. RCAN1 isoforms: RCAN1.1L (~38 kDa), RCAN1.1S (~28 kDa), and RCAN1.4 (~28 kDa). β-tubulin, loading control. **B** Densitometric measurements of RCAN1 isoform abundance normalized to β-tubulin levels are displayed as percentages of the mean relative optical density (OD) in ZT11 hippocampi. There were no temporal variations in RCAN1.1L and RCAN1.1S/1.4 levels at the time points assessed. *N* = 6 ZT11, 7 ZT17, 6 ZT23, 6 ZT5 mice. **C** Densitometric measurements of BMAL1 abundance normalized to β-tubulin levels are displayed as percentages of the mean relative OD in ZT11 hippocampi. BMAL1 levels were significantly elevated at ZT23 compared with ZT11. *N* = 6 mice per time point. **D** Representative image of the SCN (dashed white outline) in WT coronal mouse brain sections co-stained for RCAN1 (red), the nuclear marker Hoechst (blue), the clock protein BMAL1 (green), and the neuronal marker NeuN (white). RCAN1 was present throughout the SCN. RCAN1 signal specificity was confirmed with *Rcan1* KO SCN sections. Scale bar = 200 μm. **E** Higher magnification shows that RCAN1 is expressed diffusely in the SCN, with some signal colocalizing with a subset of BMAL1-positive cells (arrows). Scale bar = 20 μm. **F** Representative western blot images of RCAN1 and BMAL1 at ZT11 and ZT23 in the SCN of *Rcan1* WT and KO mice (3–6 months old) and **G** WT, *Dp16*, *Dp16/Rcan1*^*2N*^ (*D/R*^*2N*^) mice (3–6 months old). **H** Densitometric measurements of RCAN1 isoform abundance normalized to β-tubulin levels in the SCN of *Rcan1* KO mice and **I** *Dp16* and *Dp16/Rcan1*^*2N*^ mice are displayed as percentages of the mean relative OD in respective WT SCN controls at ZT11. RCAN1 levels were affected by genotype but did not differ at the time points assessed. **J** Densitometric measurements of BMAL1 abundance normalized to β-tubulin levels in the SCN of *Rcan1* KO mice and **K** *Dp16* and *Dp16/Rcan1*^*2N*^ mice are displayed as percentages of the mean relative OD in respective WT SCN controls at ZT11. BMAL1 levels in the SCN were not significantly different at the time points assessed in WT mice or compared to *Dp16* and *Dp16/Rcan1*^*2N*^ mice but were significantly elevated at ZT23 in *Rcan1* KO mice compared with WT littermates. **F**–**K** *N* = ZT11: 7 *Rcan1* WT, 5 *Rcan1* KO, 7 WT (*Dp16* strain), 6 *Dp16*, 7 *Dp16/Rcan1*^*2N*^ mice; ZT23: 5 *Rcan1* WT, 6 *Rcan1* KO, 8 WT (*Dp16* strain), 6 *Dp16*, 6 *Dp16/Rcan1*^*2N*^ mice. All data are mean ± S.E.M. *p < 0.05; ****p < 0.0001

Considering the observed impacts of RCAN1 depletion and overexpression on the periodicity and rhythmicity of circadian wheel running, we sought to determine whether *Rcan1* exhibits rhythmic expression in the light-entrained brain. To this end, we profiled the protein abundance of RCAN1 over a 24-h period in the hippocampus, a brain region that is subject to diurnal and circadian regulation and is critically involved in both memory and biological rhythmicity [23], domains which are impaired in DS, AD, and aging [67, 68]. As a reference, we also profiled the expression of the clock gene protein BMAL1 [69]. Using western blot analysis of hippocampal tissue collected at 6-h intervals from light-entrained young *Rcan1* WT mice (Fig. 10A), we found that levels of the larger RCAN1.1L protein isoform and the combined levels of the smaller RCAN1.1S and RCAN1.4 isoforms were stable across all time points assessed (Fig. 10B). Consistent with capturing rhythmic protein expression, we found significantly elevated BMAL1 expression at ZT23 (prior to light onset) versus ZT11 (prior to dark onset) (*t*(10) = −2.277, *p =* 0.046; Fig. 10C). These results imply that RCAN1 expression is normally arrhythmic in the hippocampi of light-entrained mice during early adulthood.

We next determined if RCAN1 was expressed in the SCN, the brain’s master circadian pacemaker. Consistent with a possible role for RCAN1 in influencing SCN function, we detected RCAN1 protein in the SCN (Fig. 10D). Higher magnification confirmed that RCAN1 is distributed throughout the SCN, with some signal colocalizing within the cytoplasm of a subset of BMAL1-positive cells (Fig. 10E). We then examined the rhythmicity of RCAN1 and BMAL1 levels in the SCN. Based on our hippocampal western results (Fig. 10C), we performed western blot analysis of RCAN1 and BMAL1 in the SCN of *Rcan1* KO (Fig. 10F), *Dp16*, *Dp16/Rcan1*^*2N*^ (Fig. 10G), and respective WT mice at ZT11 and ZT23.

As expected, there was a significant genotype effect on RCAN1 levels in both the *Rcan1* KO (RCAN1.1L *F*_1,19_ = 389.1, *p* < 1.0E−4; RCAN1.1S/1.4 *F*_1,19_ = 179.1, *p* < 1.0E−4) and *Dp16* (RCAN1.1L *F*_2,34_ = 14.77, *p* < 1.0E−4; RCAN1.1S/1.4 *F*_2,34_ = 13.55, *p* < 1.0E−4) strains. At ZT11 and ZT23, RCAN1.1L and RCAN1.1S/1.4 were abolished in *Rcan1* KO SCN (*p* < 1.0E−4; Fig. 10H) and increased in *Dp16* SCN (ZT11: RCAN1.1L *p* = 0.013, RCAN1.1S/1.4 *p* = 0.050; ZT23: RCAN1.1L *p* = 0.011, RCAN1.1S/1.4 *p* = 0.014; Fig. 10I) compared with WT controls. The westerns also confirmed that restoring *Rcan1* to two copies in *Dp16* mice returned RCAN1 to WT levels in *Dp16/Rcan1*^*2N*^ SCN, independent of time point assessed (*Dp16* vs. *Dp16/Rcan1*^*2N*^ ZT11: RCAN1.1L *p* = 0.050, RCAN1.1S/1.4 *p* = 0.048; ZT23: RCAN1.1L *p* = 0.039, RCAN1.1S/1.4 *p* = 0.024; Fig. 10I). In WT SCN from either *Rcan1* KO (Fig. 10H) or *Dp16* (Fig. 10I) strains, RCAN1 levels were similar at ZT11 and ZT23, consistent with our observations in WT hippocampus suggesting that RCAN1 expression is normally arrhythmic in the young mouse brain.

Unlike the hippocampus, BMAL1 levels did not differ between ZT11 and ZT23 in the WT SCN (Fig. 10J, K), consistent with previous findings [70]. However, we found a significant genotype × time interaction effect on BMAL1 levels in the *Rcan1* KO SCN (*F*_1,19_ = 4.523; *p =* 0.047; Fig. 10J). At ZT23, *Rcan1* KO mice had significantly elevated BMAL1 levels in the SCN compared with WT controls (*p* = 0.041). In contrast to *Rcan1* KO mice, we found no differences in SCN BMAL1 levels among WT, *Dp16* and *Dp16/Rcan1*^***2N***^ mice (Fig. 10K). These results may indicate a role for RCAN1 in regulating the central mammalian clock, but more studies will be required to unravel potential mechanisms underlying RCAN1 effects on molecular clock function and light-entrained diurnal and circadian activity patterns.

## Discussion

This study characterized the previously unknown impacts of RCAN1 abolition and overexpression on the periodicity, intensity, active versus inactive phase distribution, and rhythmicity of wheel running in light-entrained and free-running young versus aged mice. Using the *Dp16* mouse model for DS, we recapitulated our findings that RCAN1 modulates light-entrained diurnal and circadian locomotor activity profiles by demonstrating *Rcan1* dosage correction improved or normalized wheel running in *Dp16* mice. We also generated novel data suggesting that RCAN1 expression is normally arrhythmic in the young light-entrained brain, implying that RCAN1 expression is tightly regulated to maintain constant levels throughout early adulthood. Perturbation of RCAN1 levels early during aging, as shown using young *Rcan1* KO and *RCAN1* TG mice or *Rcan1* triplication in *Dp16* mice, disrupted light-entrained diurnal as well as circadian wheel running behavior in manners partly reminiscent of DS, AD, and aging. Taken together, these findings imply that balanced expression of RCAN1 is necessary for normal diurnal and circadian regulation of locomotor activity in mice and suggest that changes to RCAN1 levels observed in DS, AD, and normal aging [5, 7] may contribute to the diurnal and circadian dysfunctions associated with these conditions [28, 35, 40, 67].

In the DS population and DS mouse models, diurnal rest-activity and circadian-related disturbances are common, but the contribution of different loci on HSA21 has been unclear. Using RCAN1-overexpressing transgenic and *Dp16* mice, we endeavored to elucidate the contribution of *RCAN1*. In light-entrained young *RCAN1* TG and *Dp16* mice, we found that daily wheel running in the dark phase was reduced and could be normalized in *Dp16* mice by restoring *Rcan1* to two copies. These data suggest that RCAN1 overexpression may contribute to the hypoactivity in dark phase wheel running reported for the Tc1 mouse model of DS [57]. While Tc1 mice engaged in less wheel running activity during the dark phase, they were simultaneously more active in other locomotor behaviors, such as walking, climbing, feeding, and grooming [57]. Additionally, they showed increased wheel running during the light phase [57], differing from our findings in young *RCAN1* TG and *Dp16* mice. Our data also differed from previous studies that reported hyperactivity in the Ts65Dn mouse model for DS during the dark phase [55, 56] and hyperactivity of *Dp16* mice measured by the distance moved in the home cage during the light phase [58]. These differences from our findings may be explained by the activity measurement used (e.g., wheel running versus other locomotor behaviors) or suggest different interaction effects of the DS-related genes overexpressed in each mouse model. A hyperactive phenotype is more consistent with the hyperactivity characteristic of DS [39, 41]. However, an accelerated senescence phenotype is also characteristic of DS (Lott & Head, 2001; Lott, 2012), and general activity levels are well-known to decrease with aging [24, 36]. Congruent with this, we found that decreased daily wheel running in aged mice was not further reduced by RCAN1 overexpression, intimating that the hypoactivity in young *RCAN1* TG and *Dp16* mice could potentially reflect premature aging. RCAN1 overexpression is further implicated by the observation that *Dp16/Rcan1*^*2N*^ mice displayed a significant normalization of both light-entrained diurnal and circadian wheel running phenotypes compared to *Dp16* mice (Fig. 7). It remains possible that *RCAN1* TG and *Dp16* mice display hyperactivity by other measures or at earlier ages, which would be interesting to investigate in future studies.

The present study also detected attenuated rhythmicity of light-entrained diurnal wheel running as indicated by a reduced amplitude in young *RCAN1* TG and *Dp16* mice, comparable to Tc1 [57] and Ts65Dn [56] mice. Taken together with the reduced amplitudes of diurnal rest-activity rhythms reported in DS [40] and the reduced impact of the DS genotype in *Dp16/Rcan1*^*2N*^ mice, these data suggest a primary role for RCAN1 overexpression in DS-linked dampening of diurnal activity rhythms. This suggests a possible premature aging-like phenotype in young *RCAN1* TG and *Dp16* mice, as flattened rest-activity rhythmicity typically occurs with aging as well [24, 36]. While it is formally possible that RCAN1 gain of function as well as loss of function both simply result in early-age hypoactivity, a few points of evidence argue against this. Hypoactivity was not reported in earlier behavioral assessments of RCAN1-overexpressing mice [7, 18, 71] or *Rcan1* KO mice [19, 72]. Furthermore, as discussed above, DS models are generally reported as being hyperactive [58, 73–75]. Our data also indicated reduced amplitudes of light-entrained diurnal wheel running rhythms in aged mice relative to young mice. Activity rhythm amplitudes were not further reduced in aged *RCAN1* TG mice compared with NTG littermates, insinuating that RCAN1 overexpression early in life such as that found in DS [4, 5] could contribute to precocious attenuation of diurnal rest-activity rhythm amplitudes in a manner symbolic of accelerated aging. Moreover, *RCAN1* TG mice exhibited a lengthened circadian period of wheel activity, which has similarly been observed in normally aging mice [24]. This finding reveals circadian activity rhythm dysfunction in *RCAN1* TG mice which is comparable to that observed in normal aging, thereby providing further inferential evidence that RCAN1 overexpression could promote senescence-related phenotypes. As RCAN1 levels are elevated with age independent of DS [7, 8], RCAN1 may also participate in the lengthening of circadian periodicity that often manifests with normal aging. In young *Dp16* mice, interestingly, we detected no change in length of the light-entrained diurnal or circadian wheel running period whether RCAN1 was restored to disomic levels or not. This may suggest that other genes triplicated in the *Dp16* model interact with RCAN1 overexpression effects to regulate diurnal and circadian rest-activity rhythms in DS. More studies testing the contribution of different loci on HSA21 to behavioral rhythm phenotypes in DS will be important. Collectively, these results lend support to the interpretation that RCAN1 overexpression may contribute to diurnal and circadian alterations that are normal with aging but that manifest earlier in DS. Additional research is required to more fully and directly address this possibility.

A major feature of the accelerated senescence phenotype in DS is the nearly ubiquitous early-age onset of AD, which is also characterized by circadian rhythm disruptions [35]. Since RCAN1 is also elevated in AD [4–7], RCAN1 overexpression may mediate DS-AD comorbidity and link diurnal rest-activity and circadian abnormalities in both disorders. Supporting this notion, the generalized hypoactivity of daily wheel running detected in young *RCAN1* TG and *Dp16* mice mimics the increased daytime sleepiness and hypoactivity documented in AD patients [28, 31, 33]. Furthermore, the attenuated intensity and amplitude of wheel running rhythms in young *RCAN1* TG and *Dp16* mice are analogous to the fragmentation and reduced amplitude of daily rest-activity rhythms in both preclinical [36] and clinical [31] AD. The lengthened circadian period of wheel running identified in young *RCAN1* TG mice is similarly observed in mouse models of AD [37, 76]. Acrophase estimates for light-entrained diurnal and circadian wheel running rhythms did not differ between young *RCAN1* TG or *Dp16* mice and WT controls, indicating that elevated RCAN1 levels do not contribute to the circadian phase shifts observed in DS, AD, aging individuals, or animal models thereof [25, 31, 40, 56, 67]. In aggregate, these findings support the idea that RCAN1 overexpression may in part mediate overlapping aging-like disturbances of light-entrained diurnal and circadian rest-activity rhythms in DS and AD.

Importantly, circadian disruptions precede the appearance of AD-linked pathology and neurodegeneration in *RCAN1* TG mice [7], mirroring the progression of disease in AD [35, 36, 48, 67]. In a previous study, we found AD-like hippocampal mitochondrial dysfunction, oxidative stress, synaptic plasticity failure, and memory impairments in aged, but not young, *RCAN1* TG mice [7]. However, young *RCAN1* TG mice showed AD-like increases in tau hyperphosphorylation that reached the levels of aged NTG mice. This tau hyperphosphorylation was not further increased in aged *RCAN1* TG mice [7], suggesting RCAN1 overexpression accelerates tau pathology that may feedforward and contribute to AD-like phenotypes in aged mice. In the present study, we found diurnal and circadian activity rhythm alterations reminiscent of aging-associated phenotypes in young *RCAN1* TG mice. Thus, both tau pathology and diurnal as well as circadian rhythm dysfunction manifested before the development of other AD-related phenotypes in these mice, which models the preclinical, clinical, and pathophysiological characteristics of AD [35, 36, 48, 67]. Mounting evidence points to tau pathology as a more robust biomarker of AD risk than Aβ accumulation, correlating more strongly with the onset of early cognitive symptoms and eventual clinical presentation of AD [77]. Light-entrained diurnal and circadian dysfunction are also emerging as risk factors for AD, based on data demonstrating that altered behavioral rhythms precede cognitive deficits in AD [30, 32, 36] and that disrupting the circadian clockwork can drive aging-like and AD-related cognitive and pathological features [43, 44]. Taken together, our past and present data suggest that RCAN1 upregulation may promote or mediate the consequences of tau pathology and circadian dysfunction. Interestingly, the presence of tauopathy can disrupt biological rhythms [76, 78], suggesting that RCAN1 overexpression might additively or synergistically perturb the rhythmicity of activity in part through upregulating tau pathology. Given the influences of biological clocks on memory performance [23], these findings together imply that RCAN1 overexpression causes diurnal and circadian activity disruptions that may induce or exacerbate AD-related neurodegeneration.

RCAN1 deficiency altered wheel running phenotypes in the same directions as RCAN1 overexpression for some parameters but in opposite directions for others. Neither removal nor overexpression of RCAN1 affected the light-entrained periodicity of wheel running. However, the free-running periods in young *Rcan1* KO and *RCAN1* TG mice were comparably lengthened, indicating that optimal levels of RCAN1 are necessary to maintain the circadian periodicity of activity. With photic entrainment, RCAN1 removal and overexpression both reduced daily total and dark phase wheel running and attenuated the oscillatory mean (MESOR) and oscillatory range (amplitude) of diurnal wheel running rhythms in young mice. These phenotypes resemble aging, suggesting that both loss and aberrant gain of RCAN1 might accelerate aging-associated phenotypic alterations. In contrast to RCAN1 overexpression, RCAN1 abolition in young mice increased wheel running during the light (inactive) phase when mice are typically resting, reminiscent of increased nighttime awakenings/activity in DS and AD [28, 40]. Free-running *Rcan1* KO mice also showed divergent behavior from *RCAN1* TG mice. Whereas daily total and active phase wheel running and parameters of activity rhythms including MESOR and amplitude were reduced in young *RCAN1* TG mice consistently across LD12:12 and DD conditions, these measures were increased in young free-running *Rcan1* KO mice, which differed from their light-entrained counterparts. These bidirectional effects of RCAN1 downregulation and upregulation may imply that RCAN1 titrates diurnal and circadian activity levels and rhythms, which aligns with prior studies demonstrating dose-dependent regulation of locomotor activity rhythms by the *Drosophila* RCAN1 homolog *sra* [49]. The convergent effects of RCAN1 downregulation and upregulation on wheel running profiles also mirror previous findings that deletion of either *sra*, which disinhibited CaN activity, or *CanA-14F*, which encodes a catalytic subunit of CaN in *Drosophila*, both led to hyperactivity, short sleep, and arrhythmic clocks [49, 53]. Altogether, the rest-activity phenotypes with RCAN1 knockout and overexpression are complex. However, these data suggest that balanced RCAN1 expression may be required for normative light-entrained diurnal as well as circadian activity patterns and rhythms and that deviations of RCAN1 levels confer DS-, AD-, and aging-like aberrations thereof. To unravel the complex effects of deficient and excess RCAN1 levels on wheel running, future studies on the mechanism of action, such as more detailed investigation of clock gene expression, under different RCAN1 expression conditions will be needed.

Our western blot analyses revealed that RCAN1 levels are stable over a 24-h cycle in the hippocampi and SCN of young light-entrained WT mice at the time points sampled, implying that increases to RCAN1 levels as found in *RCAN1* TG and *Dp16* mice or with aging [7] and decreases to RCAN1 levels as seen in *Rcan1* KO mice could tilt the balance of RCAN1 signaling that may be regulating light-entrained diurnal as well as circadian functionality in early adulthood. Consistent with this interpretation, levels of the RCAN1.1L isoform and CaN do not fluctuate in the mouse heart [54]. CaN levels in the hamster SCN and chick retina are similarly stable [51, 52]. By contrast, the RCAN1.4 isoform exhibits circadian oscillations in the mouse heart and skeletal muscle [50, 54], demonstrating isoform-specific roles of RCAN1. Although our data suggest all RCAN1 isoforms are arrhythmic in the mouse hippocampus and SCN, it nevertheless remains feasible that fluctuations in RCAN1.4 levels are masked by RCAN1.1S or the converse, since these isoforms share the same molecular weight. Interestingly, in cases where RCAN1 or CaN levels do not show daily fluctuations, the phosphatase activity of CaN exhibits rhythmic oscillations [51, 52, 54]. Therefore, it is reasonable to infer that RCAN1 may regulate activity rhythms in part by modulating the rhythmicity of CaN activity. Since RCAN1 is known to both inhibit and facilitate CaN function [7, 59, 79] and to act independently of CaN [80], future studies are needed to determine how CaN participates in RCAN1-mediated daily activity rhythm disruptions at the crossroads of DS, AD, and aging. Moreover, it will be informative to assess rhythmic changes in hippocampal CaN activity, considering that the hippocampus contains an autonomous molecular clock that has been linked to memory performance [21–23], and since hippocampus-dependent memory deficits were previously observed in both *Rcan1* KO [19] and *RCAN1* TG [7] mice. Furthermore, profiling the rhythmicity of RCAN1-dependent modulation of CaN activity in other brain regions, such as the SCN, will be essential to establish if RCAN1 differentially regulates rhythmicity throughout the brain and to delineate the mechanisms whereby RCAN1 regulates diurnal and circadian activity patterns and rhythms.

## Conclusions

To the best of our knowledge, the present study is the first to demonstrate that both abolition and amplification of RCAN1 expression elicit an ensemble of DS-, AD-, and aging-like alterations in the diurnal and circadian patterns, periodicities, and rhythmicities of locomotion in young mice. Based on these novel findings, we posit that changes to RCAN1 levels in the brain throughout aging may perturb light-entrained diurnal and circadian activity, which could in turn contribute to aging-related cognitive impairments and/or AD progression. Accordingly, RCAN1 overexpression beginning during development in DS may be a mediator of the early appearance of circadian activity disturbances and the accelerated onset of AD- and aging-associated neurodegeneration within the DS population. In sporadic AD, RCAN1 upregulation may contribute to rest-activity rhythm anomalies that both promote and are exacerbated by AD pathology. More generally, perturbation of rest-activity profiles stemming from increased RCAN1 levels in normatively aging individuals may contribute to aging-associated cognitive decline. Future research is warranted to determine whether pharmacological targeting of RCAN1, either alone or in combination with light or melatonin therapy, may be an effective treatment for DS, AD, or aging-related phenotypes.

## Data Availability

The datasets used and/or analyzed during the current study are available from the corresponding author upon reasonable request.

## Abbreviations

DS: Down syndrome
AD: Alzheimer’s disease
RCAN1: Regulator of calcineurin 1
CaN: Calcineurin
MESOR: Rhythm-adjusted mean
SCN: Suprachiasmatic nucleus
BMAL1: Brain and muscle Arnt-like protein 1
KO: Knockout
TG: Transgenic
